# Conformational Dynamics of Intrinsically Disordered Proteins Regulate Biomolecular Condensate Chemistry

**DOI:** 10.1021/acs.chemrev.1c00774

**Published:** 2022-02-18

**Authors:** Anton Abyzov, Martin Blackledge, Markus Zweckstetter

**Affiliations:** † Translational Structural Biology Group, German Center for Neurodegenerative Diseases (DZNE), 37075 Göttingen, Germany; § Université Grenoble Alpes, Institut de Biologie Structurale (IBS), 38044 Grenoble, France; ⊥ CEA, DSV, IBS, 38044 Grenoble, France; ∥ CNRS, IBS, 38044 Grenoble, France; ‡ Department for NMR-based Structural Biology, Max Planck Institute for Biophysical Chemistry, 37077 Göttingen, Germany

## Abstract

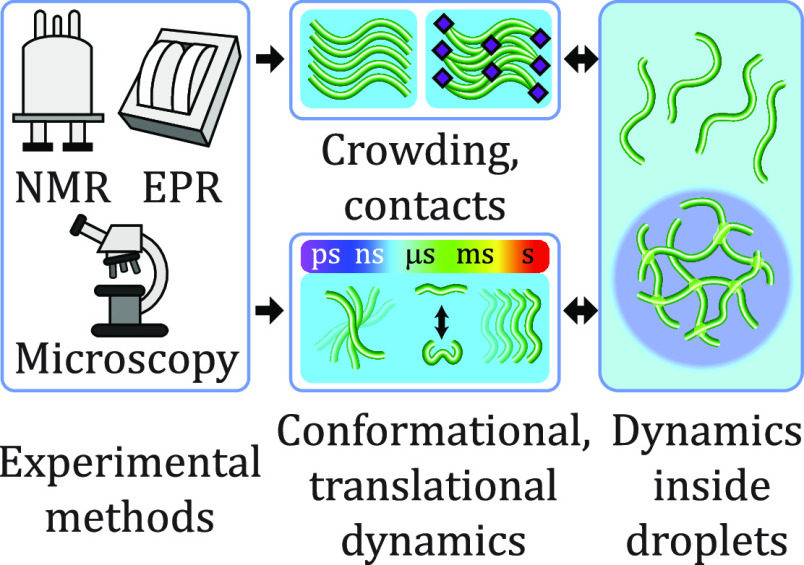

Motions in biomolecules are critical for biochemical reactions. In cells, many biochemical reactions are executed inside of biomolecular condensates formed by ultradynamic intrinsically disordered proteins. A deep understanding of the conformational dynamics of intrinsically disordered proteins in biomolecular condensates is therefore of utmost importance but is complicated by diverse obstacles. Here we review emerging data on the motions of intrinsically disordered proteins inside of liquidlike condensates. We discuss how liquid–liquid phase separation modulates internal motions across a wide range of time and length scales. We further highlight the importance of intermolecular interactions that not only drive liquid–liquid phase separation but appear as key determinants for changes in biomolecular motions and the aging of condensates in human diseases. The review provides a framework for future studies to reveal the conformational dynamics of intrinsically disordered proteins in the regulation of biomolecular condensate chemistry.

## 1 Introduction

Cells perform and control a wide range of biochemical reactions. The spatiotemporal control of biochemical reactions is realized by the internal compartmentalization of cells. Some of these compartments, such as the nucleus, endoplasmic reticulum, Golgi apparatus, mitochondria, or vacuoles, are surrounded by a lipid membrane.^1^ However, multiple biochemical reactions take place in membraneless compartments.^2−9^ Membraneless compartments, or organelles,^10^ are found in bacteria as well as human cells, both in the cytosol and in the nucleus. Because these cellular compartments are not surrounded by a membrane, they can rapidly form, change their properties, and dissolve. Nuclear organelles were the earliest membraneless compartments to be discovered. Indeed, the nucleolus^11−13^ and Cajal bodies^14,15^ were already described in the 19th and early 20th century, respectively. Later, membraneless organelles were also found in the cytoplasm. These include stress granules,^16,17^ germ granules, and P bodies.^18,19^ Reactions, which occur in and are regulated by membraneless compartments, play critical roles in diverse areas of biology such as transcription, stress response, synaptic activity, and many more.^16,20−25^ The condensation of molecules into membraneless compartments is also an important process in human diseases.^26^ Human diseases connected to membraneless compartments include cancer, neurodegeneration, and viral infections.^4,27−29^ In the case of SARS-CoV-2, liquidlike condensation of the nucleocapsid protein was suggested as a potential mechanism promoting viral genome packaging and organization of the viral replication machinery.^30−32^

Attempts to explain how these organelles exist without a membrane started to appear about a decade ago.^33,34^ Brangwynne et al. gained first insight into the physical basis of their formation in the study of germline P granules.^35^ P granules were observed to change their form upon attachment to the nucleus much as liquid drops wetting a surface. Under shear stress, P granules flowed off the nuclei, dripped, and fused into larger drops as classical liquids. Their viscosity and surface tension values were close to typical values observed in colloidal and macromolecular liquids, and their contents exist in dynamic equilibrium with surrounding liquid. Their localization behavior inside germ cells could be explained by the ability of their components to transition between a soluble form and a dropletlike condensed phase. These observations suggested that P granules form through liquid–liquid demixing.^36^ Similar properties were later observed for other membraneless compartments, including nucleoli, DNA damage repair sites, and stress granules.^10^ Based on the ability of membraneless compartments to concentrate biological molecules, Banani et al. suggested a new name, biomolecular condensates.^10^

**Figure 1:**
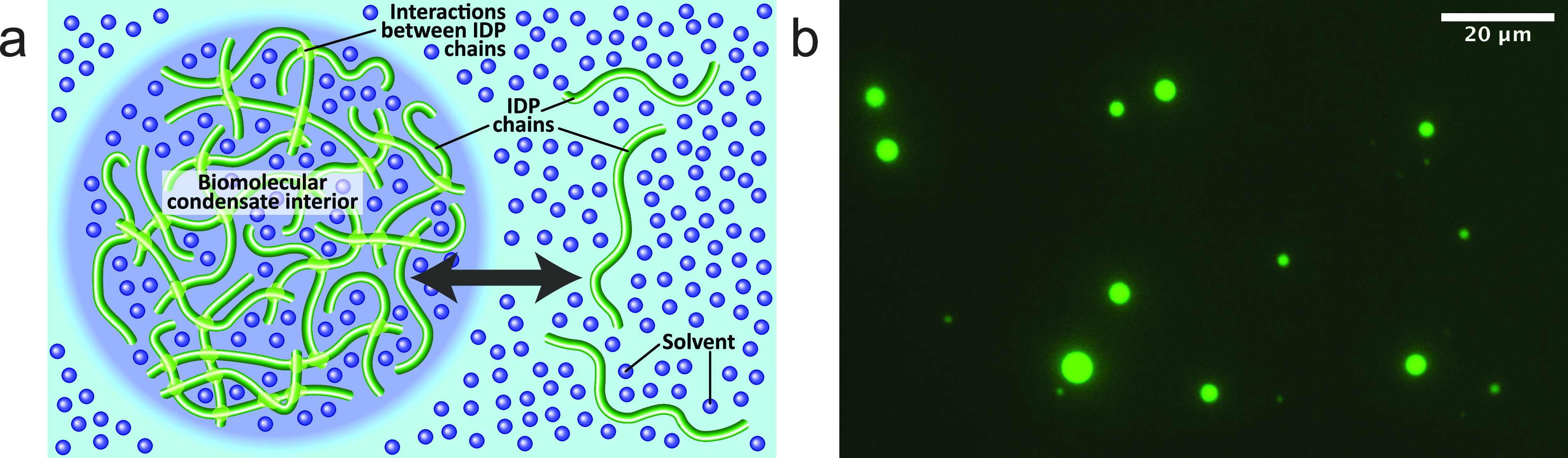
Liquid–liquid phase separation of intrinsically disordered proteins into liquidlike droplets and condensates. (a) Schematic representation of LLPS of IDPs. (b) Fluorescence micrograph of liquidlike droplets of the intrinsically disordered protein tau, which plays an important role in Alzheimer’s disease.^44−47^ In the interior of the droplets, the concentration of tau is very high. Fluorescence micrograph courtesy of Dr. Adriana Savastano [German Center for Neurodegenerative Diseases (DZNE)].

Liquid–liquid phase separation (LLPS) is the separation of molecules in solution into two phases: a condensate with high molecule concentration that often takes the form of liquidlike droplets and a surrounding diluted phase with low molecule concentration (Figure 1). This process is inherent to the thermodynamics of liquids. The theory of LLPS in polymer chainlike molecules was developed by Flory and Huggins^37−39^ and extended by Voorn and Overbeek to charged molecules.^40^ Liquids gain unique thermodynamic and other physical properties through liquid–liquid demixing. Depending on temperature and pressure, the homogeneous and phase-separated state have different free energies.^41−43^ According to thermodynamics, LLPS of macromolecules results from the interplay of the entropy of mixing, which favors a single-phase mixed state, some form of attractive interactions between molecules favoring a phase-separated state, and the configurational entropy of individual chain molecules.^37−41^

A key role in the formation and molecular properties of membraneless compartments is played by intrinsically disordered proteins (IDPs) and intrinsically disordered protein regions.^2,10,48,49^ IDPs lack a stable fold but rapidly exchange between multiple different conformations.^50−57^ Several IDPs that form membraneless compartments in living cells were also found to form aggregates in neurons affected by neurodegenerative diseases, suggesting a link between aggregation and changes in LLPS behavior.^58,59^

Interactions between IDPs and their partners involve mechanisms that exploit their high conformational plasticity,^56,60,61^ such as folding-upon-binding,^62,63^ conformational selection,^64^ fly casting,^65^ and the formation of dynamic complexes.^66^ These mechanisms are often based on the formation and stabilization of transient local structure,^62−64^ which are intimately connected to the restriction of backbone conformational sampling that occurs on the picosecond-to-nanosecond time scale.^67−69^ Secondary structure formation can be driven by sequence hydrophobicity^67^ and helix-capping interactions.^69^ In addition, local hydrophobic clusters restrict backbone motions.^70^ In agreement with the importance of transient local structures in IDPs for the molecular properties of biomolecular condensates, residual helical structures tune the phase separation of an intrinsically disordered region of the TAR DNA binding protein 43 (TDP-43).^71^ TDP-43 is present in membraneless compartments in cells, in particular in stress granules.^72^ Recruitment of TDP-43 into stress granules, together with the partially disordered protein FUS (fused in sarcoma), has been linked to pathologic aggregation in amyotrophic lateral sclerosis, a fatal neurodegenerative disease.^73^

LLPS of IDPs causes crowding of molecules and thus increased viscosity inside condensates.^74^ The increased viscosity restricts both translational diffusion and conformational dynamics of IDPs. LLPS-induced changes in the translational diffusion of IDPs can be probed by multiple methods (Table 1), including fluorescence recovery after photobleaching (FRAP),^75^ fluorescence correlation spectroscopy,^76,77^ dynamic light scattering,^78,79^ dark-field microscopy,^80^ and nuclear magnetic resonance (NMR) diffusometry.^81,82^ Reorientational dynamics on the picosecond-to-nanosecond time scale of IDPs in condensates can be studied by NMR spin relaxation,^81,83−86^ fluorescence anisotropy,^59,87,88^ and continuous-wave electron paramagnetic resonance (EPR) (Table 1).^45^ Slow conformational dynamics on the millisecond time scale can be investigated by ^15^N *R*_1ρ_ relaxation dispersion NMR.^89^ Conformational dynamics in IDPs can also be probed by photoinduced electron transfer-fluorescence correlation spectroscopy (PET-FCS)^90^ and Förster resonance energy transfer-fluorescence correlation spectroscopy (FRET-FCS),^91^ including nanosecond fluorescence correlation spectroscopy (nsFCS)^92−94^ and polarization-resolved fluorescence correlation spectroscopy (pFCS).^95−97^

**Table 1:** Biophysical Techniques That Can Be Used to Quantify Droplet Viscosity and LLPS-Associated IDP Motions

method	description	probed time scales
Methods That Probe Translational Diffusion		
fluorescence recovery after photobleaching	probes translational diffusion of fluorescently labeled IDP molecules inside droplets that generate fluorescence signal recovery; often requires cysteine residues in an IDP	milliseconds–seconds
fluorescence correlation spectroscopy	probes the translational diffusion of the fluorophore as it moves in an out of the confocal volume by analyzing its intensity correlation function	milliseconds–seconds
dynamic light scattering	probes the translational diffusion of particles in solution by analyzing the autocorrelation function of the scattered light, related to the motion of particles via the Doppler broadening	milliseconds–seconds
dark-field microscopy single plasmonic nanoparticle tracking	probes translational diffusion by directly tracking displacement of plasmonic nanoparticles embedded inside droplets	milliseconds–seconds
single-molecule fluorescence microscopy	probes translational diffusion by directly tracking fluorescent IDP molecules	milliseconds–seconds
NMR diffusometry	probes translational diffusion by spin dephasing and signal loss in the presence of a magnetic field gradient	milliseconds–seconds
single particle tracking microrheology	probes droplet viscosity by tracking positions of droplet-embedded nanoparticles; can provide information about droplet microstructure	milliseconds–seconds
Methods That Probe Reorientational Dynamics		
NMR spin relaxation	sensitive to rotational reorientation of nuclei with nonzero spin within IDP molecules. In many cases, individual values for different atoms in different residues are available.	picoseconds to ≈100 ns
fluorescence anisotropy decay	sensitive to rotational reorientation of a fluorescent dye attached to an IDP chain on the time scale of the fluorescence lifetime; generally requires cysteine residues in an IDP	picoseconds–low nanoseconds
continuous-wave electron paramagnetic resonance	sensitive to rotational reorientation of a paramagnetic spin label attached to an IDP chain; generally requires cysteine residues in an IDP	picoseconds–low nanoseconds
polarization-resolved fluorescence correlation spectroscopy	sensitive to rotational reorientation of a fluorescent dye attached to an IDP chain on time scales longer than fluorescence lifetime	nanoseconds
Methods That Probe Conformational Dynamics		
NMR relaxation dispersion	detects a contribution to NMR spin relaxation rates that is due to magnetization dephasing by interconversion between different conformations having distinct chemical shifts or transverse relaxation rates.	low microseconds–seconds
photoinduced electron transfer fluorescence correlation spectroscopy	probes contact formation (<10 Å) dynamics between the fluorophore and an aromatic residue or another quencher and the translational diffusion of the fluorophore	nanoseconds–seconds
Förster resonance energy transfer fluorescence correlation spectroscopy	probes distance fluctuations (10–100 Å) between the donor and the acceptor fluorophore in space and the translational diffusion of the fluorophore	nanoseconds–seconds

To understand the mechanisms of IDP-driven biochemical reactions inside condensates, and of the phase separation process itself, a deep understanding of the changes in the physical properties of IDPs upon LLPS is required. Because IDPs are ultradynamic proteins, detailed insight into the conformational dynamics of IDPs is particularly important. To provide a framework for future studies, we here review emerging data on the motions of IDPs inside liquidlike condensates that appeared over the past decade. First, we provide a brief overview of the current knowledge about translational dynamics of IDPs inside condensates, their viscoelasticity, and how these properties change with time after droplet formation. After that, we discuss the conformational dynamics of IDPs, how it changes inside condensates, and the methods that can be used to probe these changes, which is the main focus of this review. Finally, we discuss challenges of describing dynamics of IDPs inside condensates and possible future directions.

## 2 Dynamics of Intrinsically Disordered Proteins in Liquid–Liquid Phase Separations

### 2.1 Translational Diffusion and Viscoelasticity Inside Condensates

#### 2.1.1 Ensemble Methods

Translational diffusion is measured inside *in vitro* formed droplets and cellular condensates to probe their viscosity, which has an impact on the dynamics of IDPs. Diffusion coefficients for IDPs in droplets range between 10^–3^ μm^2^/s and 1 μm^2^/s, and droplet viscosities between 1 and 1000 Pa s according to FRAP.^75,83,98^ In comparison, a protein with a radius of gyration equal to 1 nm at 25 °C in water (viscosity coefficient 8.9 × 10^–4^ Pa s) has a diffusion coefficient of ∼250 μm^2^/s. The diffusion of IDPs in droplets is thus at least 2 orders of magnitude slower when compared to the dispersed phase. We note, however, that an accurate determination of diffusion coefficients from FRAP data can be challenging due to a strong influence of the selected model on fitting FRAP signal recovery.^75^ Often studies use simple exponential or one-dimensional (1D) models to describe diffusion inside droplets. These models, however, are in most cases not appropriate, overestimating diffusion coefficients by an order of magnitude. On the other hand, two-dimensional (2D) diffusion models with infinite boundaries were found to perform rather well, provided the size of the spot was significantly (more than three) times smaller than the droplet and the shape of the bleaching spot does not resemble a disc. In the case of oblate bleaching spots, the diffusion is effectively one-dimensional and the 1D model might be more appropriate. Taking further into account that the concentration profile of still fluorescent molecules on the border of the bleaching spot is not a perfect step function would additionally improve the precision of the extracted diffusion coefficients. Finally, for droplets that are entirely bleached, the diffusion coefficient is underestimated by an order of magnitude when estimated using infinite boundary models. Notably, the development of a finite boundary model to fit FRAP data from fully bleached droplets is not a straightforward task, likely due to the presence of an interfacial resistance in droplets that would require a careful analysis.

**Figure 2:**
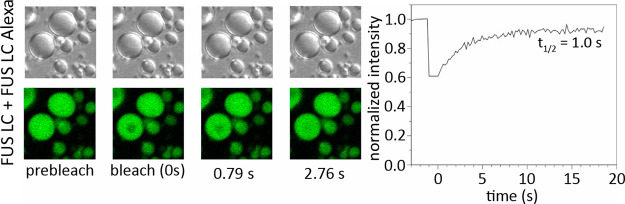
Translational diffusion in FUS LC droplets.^83^ Left panel: differential interference contrast (upper row) and fluorescence images of FUS LC droplets (lower row) before and after photobleaching. Droplets contained 0.01% FUS LC labeled with Alexa-488 at residue 86 mutated to cysteine. A 2.5 μm region inside an ∼8 μm diameter droplet was bleached. Right panel: fluorescence recovery curve after photobleaching and halftime of signal recovery. Reprinted with permission from *Molecular Cell*, Volume *60*, Issue 2, Burke, K. A.; Janke, A. M.; Rhine, C. L.; Fawzi, N. L. Residue-by-Residue View of In Vitro FUS Granules that Bind the C-Terminal Domain of RNA Polymerase II, pages 231–241 (ref (83)). Copyright 2015 Elsevier.

Diffusion of IDPs inside *in vitro* formed condensates was also measured by NMR diffusometry.^82^ For the germ-granule protein Ddx4, the translational diffusion coefficient decreased by ∼100-fold upon LLPS compared to the monomeric state.^81^ In the case of the low-complexity (LC) domain of the RNA-binding protein FUS, the LLPS-induced decrease was ∼500-fold.^82^ Notably, the translational diffusion coefficient of the FUS LC domain in the condensed phase determined by NMR diffusometry, 0.17 ± 0.02 μm^2^/s, was close to the value (0.40 ± 0.02 μm^2^/s) derived by FRAP for *in vitro* formed droplets of FUS LC (Figure 2).^83^

**Figure 3:**
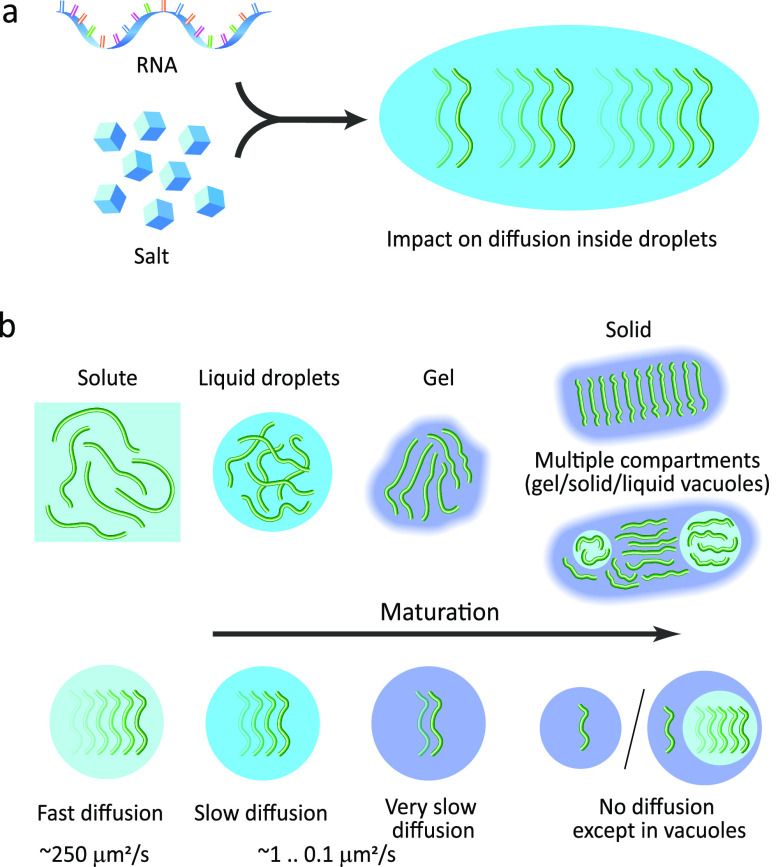
Variations in translational diffusion inside droplets due to the presence of cofactors and maturation. (a) Cofactors, such as RNA and salt, can slow down or speed up the diffusion inside droplets. (b) Translational diffusion inside droplets is attenuated when compared to the protein in the bulk solute and is further restricted upon maturation of droplets into gel-like and solid states. In some cases, multiple compartments appear in droplets upon maturation, i.e., vacuoles containing protein in the liquid state with fast diffusion, that are surrounded by the protein in the gel or even the solid-like phase.

The viscosity inside droplets/condensates differs from system to system. In addition, it can be affected by the presence of other molecules such as salt and RNA^99−101^ (Figure 3a). Brangwynne and co-workers studied protein concentration and viscosity inside droplets formed by the disordered P granule protein LAF-1 using ultrafast-scanning fluorescence correlation spectroscopy (usFCS).^102^ The calibration of the excitation volume, required for correctly estimating concentrations and diffusion coefficients, could be problematic inside droplets due to changes in the refractive index. In scanning FCS, the calibration of the excitation volume is replaced by the knowledge of the excitation path.^103,104^ Using usFCS, a droplet viscosity of 27.2 ± 5.9 Pa s (at 125 mM NaCl) was calculated from diffusion coefficients of 14 nm fluorescent spherical nanoparticles embedded into droplets. This value was in agreement with previously performed measurements based on particle tracking microrheology (see section 2.1.2.2 below). Adding short polyadenylate RNA fragments of 15 or 30 nucleotides decreased the droplet viscosity to 16.1 ± 2.8 Pa s, whereas the addition of a long 3000-nucleotide fragment resulted in a viscosity increase up to 60.9 ± 10.3 Pa s. Increasing the NaCl concentration decreased the viscosity in all cases. Based on these data, Brangwynne and co-workers suggested that the opposite-sign viscosity dependencies of LAF1 droplets with either short or long RNA fragments are biologically relevant, as RNA molecules of varying lengths are present in P granules. Variations in their relative abundance of short or long RNAs could be a natural mechanism for the regulation of droplet viscosity. Notably, the protein concentration inside LAF-1 droplets was very low, about 7 mg/mL, whereas values of ∼100 mg/mL (calculated from absorption measurements at 280 nm) are often observed in condensates formed by other IDPs.^81,83,84^ Protein concentrations inside LAF-1 droplets were calculated from FCS correlation curves. The resulting values were confirmed by calculating the concentration inside the droplets, as measured by absorption at 280 nm LAF-1 saturation concentration and average concentration in the bulk (i.e., where the mixture of monomeric and condensed phases is present), as well as measured droplet volume fraction in the bulk by three-dimensional confocal microscopy. Further diffusion experiments indicated that the LAF-1 droplets are dense when compared to the surrounding dilute phase but are nevertheless rich in solvent and full of permeable voids, with a characteristic mesh size of ∼3–8 nm, facilitating diffusion of relatively small solute molecules including folded and disordered proteins.

In many cases, droplet viscosity does not remain constant inside the condensates, but instead protein-dense droplets mature over time (Figure 3b). During the maturation process, condensates lose their liquidlike nature and form coarser gel-like structures.^105−107^ Full-length FUS droplets were found to undergo a transition to amorphous aggregates after 8 h of *in vitro* aging (shaking and mixing by pipetting).^108^ For the FUS LC domain alone, droplets were formed by cooling a disperse FUS LC solution to 4 °C; formation of gel-like assemblies was subsequently achieved by maintaining freshly formed droplets at 23 °C for 50 min.^109^ In the case of the C-terminal domain of TDP-43, incubation of liquid droplets at 42 °C for 60 min resulted in the formation of irreversible aggregates.^110^ FRAP measurements performed on droplets formed by phosphorylated full-length tau protein revealed a gradual aggregation of tau molecules inside droplets after their formation.^111^ Almost no FRAP was observed after 1 h, even when a small portion of a droplet was bleached, indicating full polymerization of tau inside droplets.^111^ In phase separation studies of the protein α-synuclein, the diffusion coefficient inside the droplets, determined by FRAP, decreased from 0.58 μm^2^/s at day 2 after LLPS to 0.23 μm^2^/s at day 5 and to 0.18 μm^2^/s at day 10.^59^ The presence of amyloid-like aggregates was detected at day 5.^59^ The increased viscosity has an impact on the reaction kinetics of proteins inside droplets and is therefore relevant for understanding the processes that lead to pathological protein aggregation.

#### 2.1.2 Single-Particle Methods

##### 2.1.2.1 Single-Molecule Confocal Fluorescence Microscopy

Single-molecule confocal fluorescence microscopy can be used to probe translational diffusion in liquid droplets. Schuler and co-workers probed IDP conformational dynamics using single-molecule fluorescence microscopy in a different but related context of molecular crowding. In the work by König et al., the IDP prothymosin α (ProTα) was fluorescently labeled at residues 1 and 56 mutated to cysteines and injected into living HeLa cells.^112^ The measured intracellular diffusion time (τ_Diff_ = 1.8 ± 0.7 ms in cytosol and τ_Diff_ = 1.6 ± 0.6 ms in nucleus) was more than 2 times larger than in buffer (τ_Diff_ = 0.7 ± 0.1 ms) and corresponds to an effective intracellular viscosity of 2.8 ± 1.1 mPa s. In a later study,^113^ König et al. repeated experiments in HeLa cells in a medium that contained 20% (w/v) PEG 400.^113^ PEG 400 does not cross the cell membrane^114^ but creates an osmotic efflux of water. This reduced the cell volume by approximately 2-fold. In this study, the diffusion time of ProTα in noncrowded cells was 1.5 ± 0.2 ms and increased to 7 ± 2 ms in crowded cells.

According to the study by König et al., the protein and nucleic acid concentrations in HeLa cells before hyperosmotic stress are 108 ± 25 mg/mL and 23 ± 5 mg/mL, respectively. Notably, HEK 293-F eukaryotic cells had similar total protein concentrations, but only 25 mg/mL were soluble cytoplasmic proteins. Taking into account protein and nucleic acid crowding, we can expect macromolecule concentrations in the cytosol of crowded HeLa cells to be on the order of 100 mg/mL or more, which is similar to values observed in IDP condensates: concentration values of 300 mg/mL and higher were reported for the intrinsically disordered hnRNPA2 low-complexity (LC) domain (determined by NMR spectroscopy)^84^ as well as for the Ddx4 LC domain^81^ and about 120 mg/mL for the intrinsically disordered FUS LC domain^83^ (determined by spectrophotometry).

##### 2.1.2.2 Single Particle Tracking Methods

Single particle tracking (SPT) methods offer an independent probe of droplet microrheology.^115^ Micro- or nanosized fluorescent beads or other observable particles (such as metal nanoparticles) are incorporated into *in vitro* formed droplets, and the trajectories of their Brownian motion-caused displacement are analyzed to probe the viscoelastic properties of the droplets. SPT was used to study droplets formed by LAF-1,^99^ yielding a viscosity value of 34 ± 5 Pa s at physiological salt conditions (125 mM NaCl). Upon addition of 5 μM RNA, the droplet viscosity decreased 3-fold to 12.8 ± 0.8 Pa s. Murakami et al. employed SPT to probe viscosities of FUS LC reversible and irreversible gels formed by cyclic cooling and rewarming.^109^ Liquid state, reversible and irreversible gel states of the FUS LC were found to have viscosities of 0.4 ± 0.03 Pa s, 3.8 ± 0.4 kPa s, and 15 ± 3 kPa s, respectively.

Liquidlike droplets and cellular condensates are not always homogeneous but can have multiple compartments^101,116,117^ (Figure 3b). Detailed insights into the microenvironment of droplets formed by the ubiquitin-binding protein p62 during their maturation were obtained by Pan et al. using SPT in combination with dark-field microscopy.^80^ Membraneless microdroplets formed immediately upon mixing p62 with GFP-labeled polyubiquitin (Ubx8). Before LLPS, gold nanorods (AuNR) were mixed with p62, and some of them were found to be embedded into p62 droplets. FRAP analysis of GFP-Ubx8 diffusion inside droplets revealed an apparent viscosity of 165.3 mPa s. The droplets fused and precipitated on the glass substrate, where they underwent a liquid-to-solid transition. During this transition, the droplets became highly heterogeneous. This included the formation of quasi-solid compartments and multiple vacuoles with external dimensions up to 18–120 μm and vacuole sizes between 0.7 and 25.6 μm.

In droplets that were undergoing liquid-to-solid transitions, the apparent diffusion rate was calculated from the Brownian motion trajectories of AuNR probes in the laboratory frame and was close to 0.3 μm^2^/s, yielding an apparent viscosity of ≈241.7 ± 157.4 mPa s in the maturing droplets. However, statistical analysis of the displacement of multiple AuNRs embedded in the same droplet revealed much lower diffusion rates relative to the droplet, ≈0.04 ± 0.018 μm^2^/s, indicating that nanorods trapped inside droplets were quasi stationary. AuNRs trapped in different regions had different diffusion rates ranging from 0.011 to 0.034 μm^2^/s, further underscoring the high heterogeneity of the droplets. A further rigidification of p62/Ubx8 droplets was observed using PEG-modified AuNRs that did not interact with p62 or Ubx8 and were mostly not trapped inside gel compartments, localizing instead in the liquid vacuoles or in the surrounding disperse phase. About 30 min after LLPS onset, PEG-AuNRs trapped inside vacuoles were repeatedly captured by the vacuole surface, suggesting that the gel phase in droplets was still fluid enough to allow for the formation of nanoscale pores or defects that captured diffusing nanorods. However, 1.5 h after LLPS, nanorods trapped inside vacuoles were experiencing elastic-like collision with their walls without sticking to them. This change in behavior suggests that at later stages of droplet maturation, the gel that surrounds the vacuoles becomes completely solid.

#### 2.1.3 Microscopic Manipulation Methods

To evaluate droplet maturation, controlled fusion events could be performed using optical tweezers or traps, where the droplets are trapped by laser beams. Freshly formed liquid droplets fuse readily into larger droplets, whereas mature gel-like droplets lose their ability to fuse.^118,119^ The droplets formed by the budding yeast translation termination factor Sup35 containing a disordered prion domain^118^ stopped fusing 1 h after LLPS. In the presence of 10% dextran, droplets formed by the FUS protein containing a low complexity (LC) domain^119^ stopped fusing after 12 h, and those formed by its G156E aggregation-associated mutant were unable to fuse already 8 h after formation. By trapping a single droplet between two optically trapped beads, Jawerth et al. studied viscoelastic properties of FUS-formed droplets and determined that at all aging steps the FUS droplets behave as a Maxwell fluid with the Maxwell relaxation time increasing with age.^120^ In addition, single-molecule manipulation methods, such as atomic force microscopy and magnetic tweezers, were proposed to study IDP molecules in liquid droplets.^121^

### 2.2 Conformational Dynamics

Conformational dynamics in IDPs occur on multiple time and length scales and are related to a complex set of processes, including the conformational sampling of individual residues, the formation of secondary structure elements, as well as large-scale conformational rearrangements of the chain. The particular physicochemical environment of membraneless compartments, as well as the presence of high concentrations of macromolecules and of intermolecular contacts stabilizing phase separated states, are expected to impact different time/length scales of IDP conformational dynamics. Changes in the conformational dynamics of polymers in the presence of crowding were both predicted in polymer physics models and observed experimentally in biomolecules (section 2.2.2). In the context of LLPS, however, the intermolecular contacts that stabilize it, specific to the sequence of each system, have an additional impact on IDP dynamics (section 2.2.3.3). Assessing experimental evidence of the impact of LLPS on IDP dynamics requires understanding of the description of their dynamics in the dilute, mixed phase. In this section, we therefore shortly review how conformational motions in IDPs can be probed and analyzed experimentally. In the end of this section, we discuss recent advances and challenges present in studies of IDP dynamics by molecular dynamics simulations.

#### 2.2.1 Reorientational Motions of IDP Chains

IDP molecules are biopolymers. Their chain dynamics can thus be partially described by models developed in polymer physics. They are however more complex than simple homopolymers, with sequence-specific effects modulating their dynamics from local backbone to long-range chain motions. Additionally, the chain contacts that stabilize LLPS in biomolecular condensates are sequence-specific. Therefore, experimental methods that probe IDP dynamics at a residue-specific level are particularly suitable for the characterization of IDP dynamics both inside and outside of membrane-less compartments. In section 2.2.1.1, we discuss NMR relaxation measurements probing fast (picosecond to nanosecond) at residue-specific level and their analysis. In sections 2.2.1.2 and 2.2.1.3, we discuss how slower chain motions (up to milliseconds) can be probed by NMR relaxometry and fluorescence spectroscopy methods.

##### 2.2.1.1 Local and Segmental Dynamics in IDPs Probed by NMR Relaxation

For a rigid protein, reorientational dynamics can be interpreted in terms of the global rotational tumbling and separate internal motions.^122,123^ This distinction starts to break with increasing flexibility in the protein chain. Already in proteins containing multiple domains connected by flexible linkers, the global tumbling rate depends on the mutual conformational arrangement of the domains.^124−126^ In intrinsically disordered proteins, there is no stable three-dimensional structure and the global tumbling appears to play little role in the description of their dynamics.^68,127−129^ Instead, their conformational dynamics can be best described in terms of motions of individual segments of the IDP chain.^68,127^ Some reference in the description of these segmental motions can be taken from models developed to study dynamics of homopolymers in the dilute limit, where intermolecular interactions play a negligible role,^130,131^ such as the Rouse model.^132^ An important definition used by these models is the Kuhn segment, a distance along the polymer chain above which the relative dynamics of two monomers is not restricted by monomers between them. According to these models, locally restricted conformational fluctuations inside each Kuhn segment form a component contributing to chain dynamics that is distinct from slower modes describing chain hydrodynamics. These slower modes describe motions in a polymer chain ranging from reorientations of individual Kuhn segments to long-range chain reconfigurations. A similar distinction between local chain dynamics and slower motions of chain segments is present in the analysis of dynamics of proteins with flexible chains, including partially folded^133^ and intrinsically disordered proteins.^67,69,134,135^

**Figure 4:**
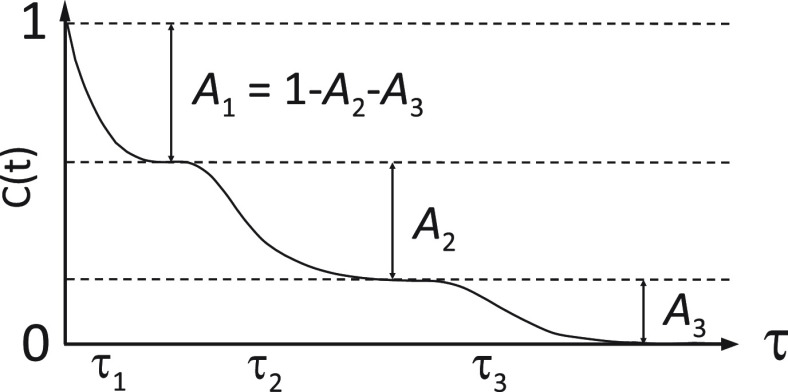
Approximation of the autocorrelation function of IDPs with three exponential components. The fastest component starts at the origin of the correlation function, where it is equal to 1.0. The intermediate and slow components start at lower levels defined by the exponential decay of the previous, faster mode. The sum of the three contributions is equal to 1.0. For example, if the intermediate motional mode has a big amplitude, its contribution to relaxation (*A*_2_) will be large. The slow component will therefore start from a level that is already close to zero (*A*_3_ = 1 – *A*_2_ – *A*_1_) and the sensitivity of the whole correlation function to slow motions will be decreased.

One of the methods of choice to study IDP dynamics is NMR spin relaxation, which is sensitive to protein motions on time scales between tens of picoseconds and tens of nanoseconds, and provides a residue-specific description thereof.^67,68,122,123,126,136−139^^15^N spin relaxation rates depend on the autocorrelation function describing the stochastic reorientation of ^15^N–^1^H bond vectors and of the closely related ^15^N chemical shift anisotropy (CSA) tensors. The “model-free” analysis, typically used in folded proteins to approximate this autocorrelation function and to obtain time scales of global tumbling and internal motions,^122,123^ is not applicable to IDPs and unfolded proteins, because these motions cannot be separated.^140^ The most successful analyses of NMR relaxation rates in IDPs were performed with an approximation of the autocorrelation function as a sum of three exponentially decaying components (Figure 4)^69,131,135^ that was first proposed by Brutscher et al.^133^ Each of the three components has a characteristic time τ_*k*_, which represents the time scale of a distinct motional mode, and the amplitude *A*_*k*_ representing the contribution of the motional mode to the relaxation rate:^131,135^
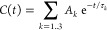
When the amplitude of the slowest component (*A*_3_) is small, the chain is very flexible. In this case, the more local motions have large amplitudes (*A*_1_ + *A*_2_), which efficiently quench the autocorrelation function. For large *A*_3_ values, on the other hand, local motions are more restricted and chain segmental motions significantly contribute to the measured relaxation rates. Therefore, it is related to the local backbone conformational entropy.^141^ This relationship is however not straightforward, in particular because of the collectivity of motions of different residues and because not all chain motions are probed by NMR relaxation.^142^ Importantly, a faster mode decays fully before a slower mode starts, if the three characteristic times τ_*k*_ are sufficiently separated^126,143^ (Figure 4). In such a situation, the three components describe statistically independent motions, validating the three-component approach. Otherwise there is no guarantee that the fitted time scales represent distinct motional modes.

The three-component analysis of conformational dynamics is also applicable to unfolded proteins, as shown earlier by Skrynnikov and co-workers.^144^ They used ^15^N NMR spin relaxation measurements to rescale MD simulations and thus extract from the MD trajectories correlation functions, which describe the reorientation of the backbone ^15^N–^1^H bond vectors of urea-unfolded ubiquitin. Three exponential components, with average (over the protein sequence) correlation times of 44 ps, 1.4 ns, and 9.4 ns and weights of 30%, 42%, and 28%, respectively, were identified. The shortest 44 ps component was assigned to fast peptide plane librations. The ∼1 ns component was attributed to local changes in the peptide plane dihedral angles. These might be compensated by motions in adjacent residues and therefore do not impact the global shape of the chain. The more long-range motions, which involve the conformational rearrangement of chain segments, were best described by the ∼10 ns component. Skrynnikov and co-workers also pointed out the role of the resistance of the solvent to the long-range chain conformational rearrangements (“hydrodynamic drag”) in the separation between local and segmental motions. Notably, correlation functions obtained from MD simulations performed in vacuum (i.e., without solvent) could be described with only one exponential component, and local changes in individual dihedral angles were resulting in global conformational rearrangements, highlighting the importance of the solvent for IDP dynamics.^144^

**Figure 5:**
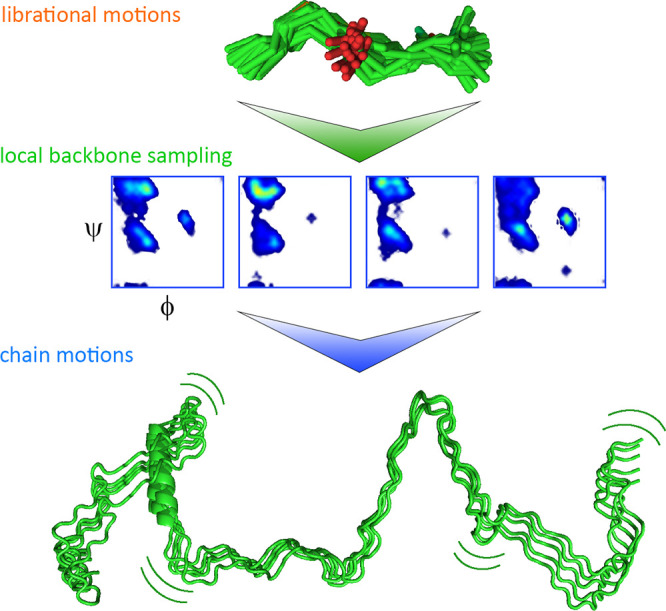
Physical origin of picosecond-to-nanosecond time scale motions in IDPs. The fastest mode reports on the librational motions of the ^15^N–^1^H bond vector, the intermediate mode reports on the local backbone dihedral angle sampling, and the slow mode reports on the chain motions.^135,144^ Adapted with permission from the *Journal of American Chemical Society*, Volume *138*, Issue 19, Abyzov, A.; Salvi, N.; Schneider, R.; Maurin, D.; Ruigrok, R. W. H.; Jensen, M. R.; Blackledge, M. Identification of Dynamic Modes in an Intrinsically Disordered Protein Using Temperature-Dependent NMR Relaxation, pages 6240–6251 (ref (135)). Copyright 2016 American Chemical Society.

To gain further insight into the physical origin of fast, intermediate, and slow components of the autocorrelation function of IDPs, Blackledge and co-workers analyzed characteristic times and amplitudes of three components in the C-terminal domain of the nucleoprotein of Sendai virus (N_TAIL_).^135^ Dynamic parameters were fitted to 58 relaxation rates measured at four different magnetic fields (14.1–22.3 T) and four temperatures (274–298 K). The observed separation of the extracted time scales supported the independence of the associated motional modes. In addition, the temperature and sequence dependence of their time scales pointed to distinct physical origins, as previously suggested by polymer models. The fastest of the three modes, which had a time scale of tens of picoseconds and exhibited a negligible temperature dependence, was assigned to librational motions of the ^15^N–^1^H bond vector and the CSA tensor (Figure 5). The dominant contribution to the autocorrelation function came from the intermediate mode with a time scale of about 1 ns. Its flat sequence profiles of both time scale and amplitude (except in the helical region) suggested that it is independent of the chainlike nature of the protein. Supported by its temperature dependence, it was assigned to local backbone sampling (Figure 5). The characteristic times of the slow mode varied between 5 and 25 ns along the N_TAIL_ sequence. Both the sequence profiles of its amplitude and time scale were bell-shaped (decreasing toward the chain termini), implying that it is related to chainlike or segmental motions. Its temperature dependence correlates with the temperature dependence of the solvent viscosity, indicating that its dynamics are coupled to the solvent, as expected for chain motions. Therefore, it was assigned to the motions of the N_TAIL_ chain (Figure 5).

While basic relaxation properties of IDPs can be related to those identified in homopolymers (i.e., the presence of local and segmental motions), multiple features of IDP dynamics originate from the specific properties of residues in their chains. Residual secondary structure elements can result in a significant deviation of the dynamic parameters from values observed in the highly flexible parts of the chain. In addition, sequence hydrophobicity^67,145^ and the presence of charged residues^146^ influence chain conformational dynamics.^135^ Differences in amino acid bulkiness can further influence IDP dynamics, resulting in deviations from random coil-like chain behavior.^147^ Importantly, the presence of intermolecular contacts, such as in the condensates formed by LLPS, will likely influence the description of motional modes developed for IDPs in the dilute limit.

##### 2.2.1.2 Segmental Dynamics in IDPs Probed by NMR Relaxometry

Parigi et al. used field-cycling ^1^H NMR relaxometry to study long-range segmental dynamics in IDPs. Dispersion profiles of collective, nonresidue specific proton relaxation rates were acquired at 298 K at fields ranging from 0.02 to 45 MHz for four IDPs: the 140-residue protein α-synuclein, the 134-residue β- synuclein, a 99-residue fragment of the protein Tau termed K19, and the 129-residue protein lysozyme in a denatured state. The dispersion profiles were fitted with a collective correlation time τ_R_ describing correlated motions of IDP segments, associated squared collective order parameter *S*_C_^2^, representing the amplitude of contribution of these motions to the relaxation, and an additive parameter α representing the residual contribution of faster motions. τ_R_ values of 7.9 ± 0.5 ns, 6.5 ± 0.3 ns, 6.1 ± 0.9 ns, and 5.3 ± 0.4 ns were obtained for α-synuclein, β-synuclein, denatured lysozyme, and Tau K19, respectively, with associated order parameters being close to 0.1. This range of τ_R_ values (6–9 ns) is similar to the time scale of long-range segmental motions determined in unfolded ubiquitin by Skrynnikov and co-workers (*vide supra*).

**Figure 6:**
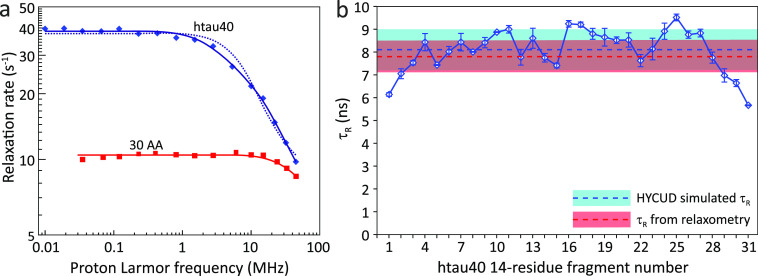
IDP chain motions probed by NMR relaxometry.^128^ (a) Protein proton relaxation rates are dependent on IDP chain length. Collective rates of a 30-residue N-terminal fragment of α-synuclein (red squares) and of 441-residue human tau protein htau40 (blue diamonds). The fit with one correlation time for htau40 is shown as a dotted line and with two correlation times as a solid line. (b) τ_R_ values predicted by HYCUD for the 441-residue htau40 protein. An atomic effective radius (AER) of 3.3 Å was used. Blue line with diamonds: τ_R_ values predicted for different 14-residue fragments. Dashed blue line and blue box: τ_R_ averaged over all fragments and standard deviation of the average value. Dashed red line and red box: τ_R_ calculated from relaxation dispersion data and standard deviation of the average value. HYCUD predicts a bell-shaped sequence profile of τ_R_ values, with the average predicted τ_R_ value matching that obtained experimentally. Adapted with permission from ref (128). Copyright 2014 American Chemical Society.

To evaluate the impact of the chain length on segmental dynamics in IDPs, Parigi et al. also measured proton longitudinal relaxation dispersion in a fragment comprising the N-terminal 30 residues of α-synuclein (named N30 α-synuclein) and in htau40 the longest isoform of Tau with 441 residues^128^ (Figure 6). In the case of N30 α-synuclein, it was possible to estimate only the upper (slower) limit of τ_R_, which was around 2 ns. For htau40, the dispersion profiles were initially fitted with a single τ_R_, resulting in τ_R_ = 10 ns and *S*_C_^2^ = 0.12. However, this fit was not optimal, and it was repeated with two correlation times, τ_R1_ and τ_R2_, resulting in τ_R1_ = 27 ± 0.5 ns, *S*_C1_^2^ = 0.02 and τ_R1_ = 4 ns, *S*_C2_^2^ = 0.17. Based on the differences in the τ_R_ values of N30 α-synuclein, α-synuclein, and htau40, it was concluded that the correlation times of segmental motions in IDPs increase with the length (or molecular weight) of the protein, and that in IDPs with long chains (such as htau40), additional modes with slower correlation times may be necessary to describe their dynamics. Notably, the chain length dependence of segmental motions is not usually detectable by high-field NMR relaxation experiments in unfolded proteins/protein regions, as the autocorrelation function is already quenched by faster local motions. Field-cycling ^1^H NMR relaxometry, however, probes lower frequencies and can therefore be more sensitive to slower chain motions that may appear in longer chains. Indeed, the combination of lower field measurements using NMR relaxometry and residue-specific high-field NMR relaxation experiments are likely to be particularly powerful to dissect IDP motions on multiple time scales.^148−151^

The intrinsic dynamics of IDP chains can also be predicted from the amino acid sequence of an IDP taking into account hydrodynamic coupling.^128^ This approach is based on the HYCUD (hydrodynamic coupling of domains) algorithm.^124,152^ In HYCUD, an IDP chain is split into multiple rigid nonoverlapping fragments of the size of the Kuhn segment, 14 residues.^70,127^ As part of the IDP, each fragment is experiencing an increase in the effective viscosity relative to the viscosity of the solvent, due to the presence of other fragments. This effective viscosity is calculated from the intrinsic viscosity of all other fragments, calculated by HYDROPRO,^153^ and their effective concentration calculated based on the distance to the fragment in question. The rotational correlation time of the fragment in question is then slowed according to the ratio between effective and solvent viscosities. HYCUD was shown to accurately predict τ_R_ values in different IDPs. In addition, the experimentally observed dependence of τ_R_ on IDP chain length was well reproduced. Future work has to show to which degree the residual structure, which might change the persistence length,^154^ influences the HYCUD-based chain dynamics description of IDPs.

##### 2.2.1.3 Slow and Long-Range Chain Conformational and Reorientation Dynamics Probed by Fluorescence Spectroscopy Methods

**Figure 7:**
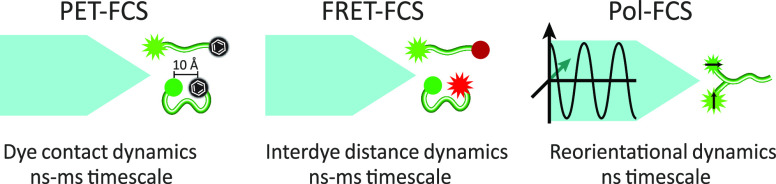
Variants of fluorescence correlation spectroscopy. PET-FCS (left panel) probes chain conformational dynamics through the contact (distance <10 Å) formation rate between a fluorophore and a quencher (typically, an aromatic residue or another dye). FRET-FCS (middle panel) describes chain conformational dynamics by correlating the intensities of donor and acceptor fluorophores. Depending on the distance between dyes (should be in the 10–100 Å range), FRET between donor and acceptor dyes occurs more or less efficiently, and we observe acceptor instead of donor fluorescence. Polarization-resolved FCS (Pol-FCS, right panel) probes chain reorientational dynamics by correlating donor fluorescence after excitation by polarized light. The efficiency of photon absorption by the donor depends on its orientation relative to the light polarization direction.

Slower and longer-range chain conformational dynamics in IDPs, and their changes upon LLPS, can be accessed using fluorescence spectroscopy methods (Figure 7), such as fluorescence correlation spectroscopy (FCS), which is sensitive to chain motions on timescales slower than the fluorophore lifetime (several nanoseconds). Originally FCS was designed to probe translational diffusion of the fluorescent molecule, as it moves in and out of the measurement volume.^76,155^ If the IDP chain contains an attached fluorophore and quenchers (such as aromatic residues), contacts between the quenchers and the fluorophore result in fluorescence quenching through photoinduced electron transfer (PET), and the time scale of the quenching process is reported by the fluorescence intensity correlation function. PET-FCS uses this effect to probe chain reconfiguration events resulting in these contacts on time scales between nanoseconds and milliseconds.^90^ Alternatively, FCS can be combined with Förster resonance energy transfer (FRET). If the protein is labeled with a donor and an acceptor fluorophore, the variations of distance between the two probes would result in variations of the efficiency of an energy transfer between them, which are reported by the donor and acceptor intensity autocorrelation functions and the cross-correlation between donor and acceptor intensities.^91^

**Figure 8:**
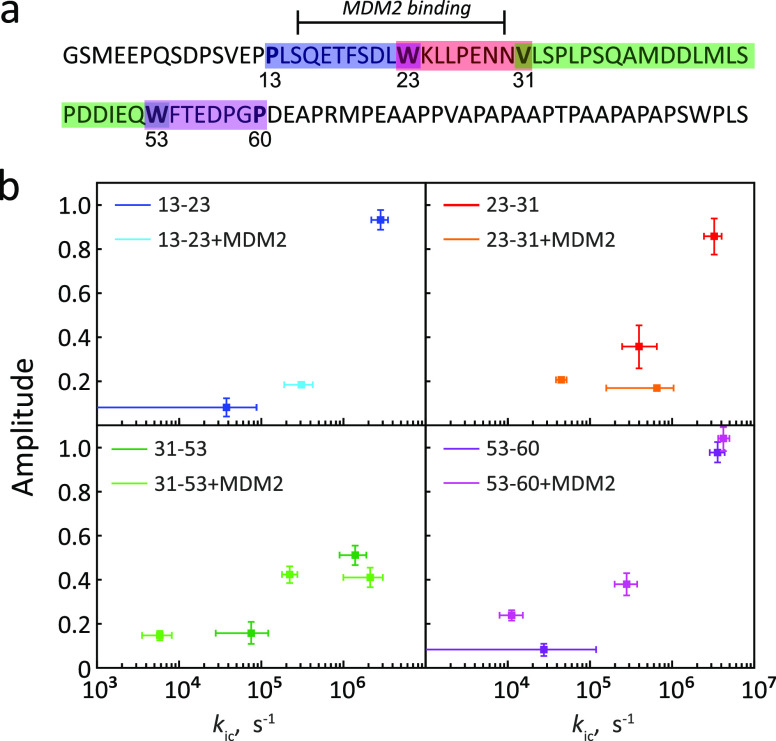
Chain dynamics of the disordered N-terminal TAD domain of p53 probed by PET-FCS.^156^ (a) Sequence of the N-terminal TAD domain of p53. Color bars: four regions (“loops”), of which the dynamics were probed. Each loop contains a tryptophan on one end and a fluorescent dye on the other end. The first two loops (13–23 and 23–31) comprise the MDM2 binding site. (b) Rates of loop closure for each loop, in free p53-TAD (darker colors) or with bound MDM2 (lighter colors). The number of kinetic components varied depending on the loop and was influenced by MDM2 binding. Adapted with permission from ref (156). Copyright 2011 American Chemical Society.

PET-FCS was used to probe chain dynamics of the disordered N-terminal TAD domain of the tumor suppressor protein p53.^156^ p53-TAD natively contains three tryptophan residues, and they were mutated to phenylalanine residues, leaving alternatively tryptophan at position 23 or 53. Residues 13, 31, and 60 were alternatively mutated to cysteines to attach a fluorescent oxazine dye. In this way, it was possible to study the time scales of loop closure for the chain loops 13–23, 23–31, 31–53, and 53–60 of p35-TAD. For all four chain loops, FCS had a fast component, assigned to loop closure kinetics (corresponding to the time scale of ∼100 ns), and a slower microsecond component of minor amplitude, assigned to larger-scale chain conformational reorganizations having an impact on loop closure kinetics (Figure 8). Upon binding of MDM2, the fast ∼100 ns loop-closing component slowed down to the microsecond time scale in the loop 13–23 involved in the binding interface.

**Figure 9:**
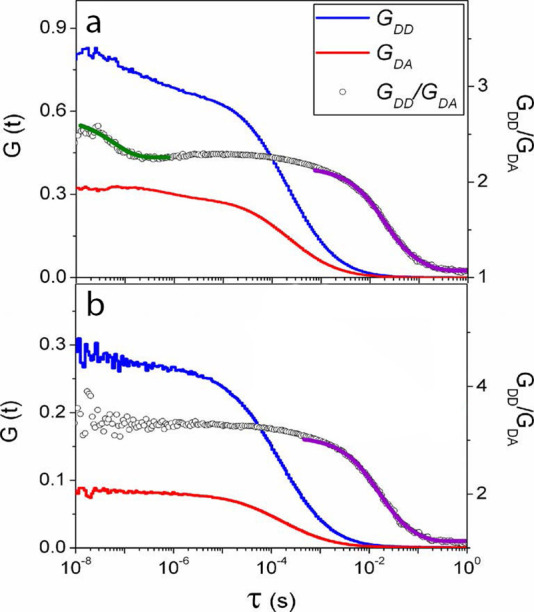
FRET-FCS fluorescence correlation curves describing dynamics of the disordered cyclin-dependent kinase inhibitor Sic1^157^ in Tris buffer (a), pH 7.4, and in Milli-Q water (b). The green line indicates an exponential decay fit in the nanosecond range; purple lines in the millisecond range. Adapted with permission from the *Journal of Physical Chemistry B*, Volume *118*, Issue 15, Liu, B.; Chia, D.; Csizmok, V.; Farber, P.; Forman-Kay, J. D.; Gradinaru, C. C. The Effect of Intrachain Electrostatic Repulsion on Conformational Disorder and Dynamics of the Sic1 Protein, pages 4088–4097 (ref (157)). Copyright 2014 American Chemical Society.

FRET-FCS was used to probe chain conformational dynamics in the 283-residue disordered cyclin-dependent kinase inhibitor Sic1 (Figure 9).^157^ Measurements were performed in Tris buffer (150 mM NaCl, 50 mM Tris at pH7.4) and in pure Milli-Q water to evaluate the impact of charge screening on chain dynamics. In Tris buffer, a nanosecond-time scale component and two millisecond-time scale components (17.3 ± 0.7 ms and 83.7 ± 6.3 ms) to FCS were observed. In pure water, however, only millisecond-time scale components (13.1 ± 0.9 ms and 58.6 ± 6.1 ms) were detected, and the nanosecond-time scale component disappeared. From these data it was concluded that the Sic1 chain was stiffer in pure water compared to Tris buffer due to electrostatic repulsion between positively charged residues in the absence of charge screening, and this led to the disappearance of fast nanosecond-time scale dynamics.

However, in both PET-FCS and FRET-FCS, probe chain conformational dynamics are limited to the relative translational diffusion of two positions in the IDP chain. Additional insight can be gained when measuring FCS in polarized light (polarization-resolved FCS; Pol-FCS),^158^ because the efficiency of dye excitation by polarized light depends on its constant fluctuating orientation in space, and the correlation curve thus captures these fluctuations. Indeed, Pol-FCS was used to understand whether heterochromatin protein 1 (HP1) undergoes LLPS in cells.^159^ To this end, green fluorescent protein (GFP) was covalently attached to HP1. The hypothesis underlying the experiments was that the presence of LLPS in chromocenters would result in increased local viscosity of the medium and decreased rotational diffusion through protein–protein interactions. Translational diffusion would be, on the other hand, impacted by factors such as binding interactions with chromatin and collisions with diffusion barriers and would be a less precise probe of LLPS. However, the GFP-HP1 rotational correlation time observed in chromocenters (111 ± 8 ns) was similar to that in the surrounding nucleoplasm (117 ± 9 ns), and only in the cytoplasm it was smaller (74 ± 7 ns). This suggested that there might not be a specific viscous microenvironment surrounding chromocenters. The combination of polarization-resolved FCS with fluorescence anisotropy measurements (discussed in section 2.2.4.1) could potentially probe chain reorientation dynamics on timescales from 0.1 ns to milliseconds.^160^

In filtered FCS, signals emanating from different fluorescent species present in solution can be detected and processed separately, based on their properties such as fluorescence lifetime and polarization-resolved spectral information.^161^ These species could also represent the same molecule but in different conformations modulating fluorescence parameters,^161^ allowing a more precise description of conformational and reconfiguration events governing chain dynamics. Tsytlonok et al. used filtered FCS to study conformational dynamics of the intrinsically disordered cyclin dependent kinase inhibitor protein p27. Fluorescence signals were filtered based on observed FRET efficiencies and fluorescence autocorrelation curves. In addition, cross-correlation curves were analyzed with four exponential decay components describing chain dynamics. The three slowest components (2–2.6 μs, 23.1–25.7 μs, and 297–363 μs) had similar time scales among different constructs. In contrast, the shortest component was significantly different with relaxation times of 22 ± 30 ns, 70 ± 40 ns, and 130 ± 30 ns for the donor/acceptor pairs 29/54, 54/93, and 75/110, respectively. Tsytlonok et al. concluded that the faster 22 ns-time scale dynamics occurring in the p27 region between residues 29–54 is important for p27’s ability to undergo chain expansion in the interaction with Cdk2/Cyclin A.

Chain distance dynamics that occur on time scales slower than milliseconds can be probed by analyzing single-molecule Förster resonance energy transfer (smFRET) efficiency histograms (time scales up to 0.1 s) and total-internal reflection fluorescence (TIRF) intensity trajectories of immobilized molecules (time scales up to 1000 s).^88^ On the opposite side, a more detailed description of chain reorientation dynamics on the nanosecond timescale could be obtained using nanosecond FCS-FRET (nsFCS). nsFCS is based on the detection and analysis of donor and acceptor intensity fluctuations on the timescale of their lifetimes and is sensitive to chain motions in the 10–1000 ns range.^162^ Donor and/or acceptor intensity correlation functions can be fitted with a model of diffusion in a potential of mean force describing the random displacement of one probe relative to another with an equilibrium distance between them.^92−94^ The relaxation time, τ_r_, obtained from this model, corresponds to the reconfiguration time of the chain between the two fluorophores. In addition, the distance distribution function can be extracted from the smFRET transfer efficiency histograms, and the radius of gyration (*R*_g_) of the chain can be estimated. nsFCS is complementary to the “classic” FRET-FCS, and the two methods can be combined, potentially allowing one to observe motions on time scales between nanoseconds and seconds.^94^ Rezaei-Ghaleh et al. used nsFSC to probe long-range chain dynamics in α-synuclein, fluorescently labeled at residues 42 and 92 mutated to cysteines. When using a single-exponent model for fitting the nsFCS data, a τ_r_ of 58 ± 13 ns was obtained. However, additional MD simulations suggested that a double-exponent model explains better the distance correlation functions obtained from the trajectory. Fitting also the nsFCS experimental data with a double-exponent model yielded a shorter correlation time τ_r1_ = 23 ± 4 ns (contributing 66 ± 2%) and a longer correlation time τ_r2_ = 136 ± 33 ns (with a contribution of 34 ± 2%), potentially probing two modes in the spectrum of chain relaxation modes predicted by the Rouse model. In addition, a combination between nsFCS and PET-FCS was proposed by Schuler and co-workers.^163^

Time scales probed by fluorescence correlation spectroscopy methods provide information about longer-range chain reconfiguration modes than those probed by NMR relaxation and relaxometry. Because the current data suggest that the conformational dynamics of IDPs in condensates are slowed down (reviewed in section 2.2.3.1), FCS experiments could be particularly useful to interrogate the slowed down motions and thus shed light on the changes in the dynamic properties of IDPs upon phase separation. However, quantitative analysis of time scales and rates extracted from FCS analysis should be done with care. First, the time scales obtained in different methods are not equivalent. PET-FCS reports only on the formation of contacts shorter than 10 Å, whereas FRET-FCS reports on events where dyes are in a distance range between 10 and 100 Å, and a conversion is required^164^ to compare values from two methods. Polarized FCS probes rotational reconfiguration of chain segments and not their relative translational diffusion, and though the two are related to the chain reconfiguration dynamics, a chain model would be required to reconcile the two values. Importantly, as in fluorescence anisotropy measurements described later in this review, the fitted time scales are likely representing averaged values over the timescale range to which the method was sensitive in a given system.

#### 2.2.2 Dependence of the Experienced Viscosity on the Probed Length Scale

An important property of the physicochemical environment inside membraneless compartments is the presence of macromolecular crowding, i.e., high concentrations of macromolecules that result in high viscosities and slower dynamics. Both translational diffusion and chain motions become slower with increased solvent viscosity. If the diffusion (translational or rotational) was explained only by the Stokes–Einstein equation, diffusion times should increase by the same factor with increased solvent viscosity irrespective of the size of the diffusing molecule. However, in condensates formed by the LC domain of FUS, protein molecules diffused 500-times slower than in the dispersed phase, and for the smaller buffer molecules the diffusion was only 6-fold slower.^82^

In fact, it was suggested that in crowded environments formed by polymer solutions the viscosity does not have a uniform value but rather depends on the size (length) scale on which it is probed, i.e., small probes, such as water molecules, experience a smaller impact on their diffusion than larger probes such as proteins. This effect was observed both in solutions of crowding agents and in the cytoplasm of living cells.^165−168^ In a basic description of the length-scale viscosity model,^167^ the motion of a probe particle of radius *r*_p_ inside the polymer solution generates a hydrodynamic flow with an effective length scale of *R*_eff_ that also depends on the hydrodynamic radius of the polymer molecule *R*_h_. If *r*_p_ ≪ *R*_h_, then *R*_eff_ ≈ *r*_p_, and if *r*_p_ ≫ *R*_h_, then *R*_eff_ ≈ *R*_h_. This flow would be screened at a distance ξ, which is the characteristic size of “openings” in the polymer mesh (distance between a monomer of one chain and the nearest monomer of another chain). The viscosity experienced by the probe then depends on the ratio between flow size and the size of mesh “openings” (*R*_eff_/ξ). A small flow created by small molecules will hit into an obstacle at a distance much larger than its characteristic size; therefore, the experienced viscosity would be similar to that of the solvent. Inversely, a large flow created by objects with size much larger than ξ is immediately impacted by the polymer mesh, and the object experiences the macroscopic viscosity of the polymer. The alternative explanation of this effect was related to the presence of a depletion layer surrounding a particle in a polymer solution, in which the concentration of polymer monomers is reduced,^167^ because it cannot sample conformations that experience a steric clash with the particle, so its entropy in its vicinity is reduced. This concentration gradient of polymer monomers near the probe particle creates an effective viscosity gradient that spans from the solvent viscosity to the macroscopic viscosity of the polymer. Notably, the models developed to explain the depletion layer effect were shown to be related to the length-scale model.^167^ The dependence of the viscosity experienced by the probe on its size in polymer solutions or crowded cellular environment can be evaluated by measuring the translational diffusion of probes of different size, such as biomolecules,^166^ nanodiamonds,^166^ or polymer nanoparticles.^169^ However, the length-scale effect does apply not only to molecules of different sizes but also to different motions of the same molecule happening on different length scales.

Rezaei-Ghaleh and co-workers proposed ^17^O NMR *R*_1_ relaxation spectroscopy^170^ and more recently ^23^Na NMR chemical shift, *R*_1_ relaxation, and pulse field gradient diffusion spectroscopy^171^ to probe solvent properties in phase-separating systems. Notably, in water–glycerol mixtures of different viscosities, relative ^17^O and ^23^Na *R*_1_ rates (ratio of *R*_1_ rate measured in water–glycerol mixtures to the rate measured in water) scaled differently with the viscosity of the mixture. The relative viscosity of a 200 mg/mL glucose solution to water was calculated on the basis of relative ^17^O and ^23^Na *R*_1_ rates, and different values (1.70 ± 0.09 for ^17^O and 2.28 ± 0.09 for ^23^Na) were obtained. A similar difference in relative viscosities (2.01 ± 0.10 for ^17^O and 3.85 ± 0.06 for ^23^Na) was observed in 200 mg/mL Ficoll solution. Some of this difference in relative viscosity experienced by sodium ions and water molecules could arise from the fact that due to their different size they probe solvent viscosity on different scales.

**Figure 10:**
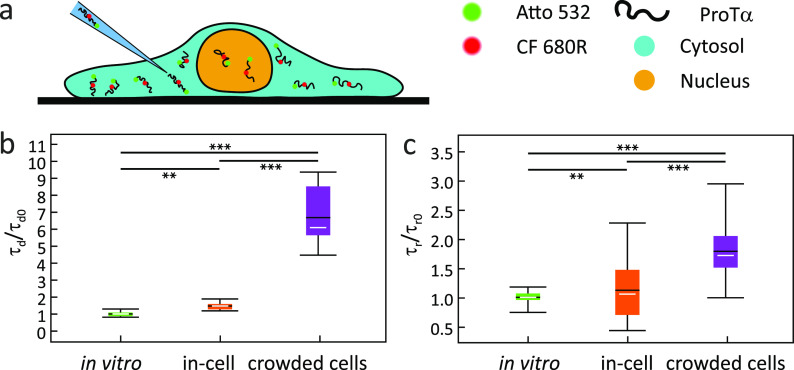
IDP dynamics probed in living cells by nsFCS. (a) Illustration of a HeLa cell with injected fluorescently labeled ProTα. (b) Relative diffusion times (τ_d_) and (c) relative τ_r_ values obtained in buffer, in HeLa cells cytosol without and with hyperosmotic stress.^113^ The fence indicates the total range of τ_r_ values, the white line indicates the median value, the black line indicates the mean value, and the colored box indicates the range between the first and the third quartile. The statistical significance of differences between mean values was verified by the Kolmogorow–Smirnow test (**P < 0.01, ***P < 0.001). Adapted with permission from ref (113). Copyright 2021 John Wiley & Sons.

Schuler and co-workers observed that the translational diffusion of the intrinsically disordered protein ProTα in crowded HeLa cells (Figure 10) is much more slowed down (7.6×) than its chain reorientational dynamics measured by nsFCS (2×), compared to the buffer. They concluded that translation diffusion and chain reorientational dynamics of ProTα in crowded cells act as viscosity probes of different length scales. For translational diffusion, the relevant scale is in the micrometer range, above the characteristic length of intracellular crowders, whereas for the chain reorientation it is in the low nanometer regime, i.e., below this length.^113^ To further probe the length scale effects on crowder viscosity and molecular diffusion, the small fluorophore Atto 532 (molecular weight MW = 0.9 kDa; hydrodynamic radius *r*_h_ = 0.5 nm) and the folded protein β-glucuronidase (MW = 280 kDa; *r*_h_ = 5.1 nm) were injected into crowded HeLa cells. Surprisingly, the relative diffusion time of ProTα (MW = 12 kDa; *r*_h_ = 4.4 nm) in crowded cells was faster than for the folded protein β-glucuronidase of almost the same hydrodynamic radius and close to that of a very small molecule Atto 532. These results suggest that with respect to the translational diffusion of ProTα, the effective viscosity probe size is much smaller than its hydrodynamic radius, on the order of the Kuhn segment length.

Not only translational diffusion and chain reconfiguration dynamics can be associated with viscosity probes of different sizes, but different chain dynamic modes themselves can be represented as probes of different sizes. Adamski et al. used ^15^N relaxation and the three-component autocorrelation function analysis to study the crowding and viscosity dependence of the conformational dynamics in two IDPs in different concentrations of dextran40.^172^ Both the slowest and the intermediate time scale component displayed a linear dependence on solvent viscosity as probed by proton longitudinal NMR relaxation. Notably, the slowest component, describing chain motions, displayed a stronger viscosity dependence than the intermediate component, which describes local backbone sampling. These observations suggested that local backbone dynamics can be associated with a probe of the size of an individual amino acid, while segmental dynamics are effectively a probe of the size of several residues.^172^ Interestingly, component amplitudes did not experience any significant changes with increasing crowding agent concentrations, at least for the dextran concentrations below 145 mg/mL, indicating no significant impact on local backbone conformational entropies. At dextran40 concentrations above 145 mg/mL, the solvent viscosity deviated from a linear behavior, indicating a transition to the semidilute regime, where protein and dextran molecules start to interact, penetrating their respective hydrodynamic volumes. This regime may be particularly relevant for condensates formed by IDPs,^172^ where IDP concentrations can reach 100–400 mg/mL.

**Figure 11:**
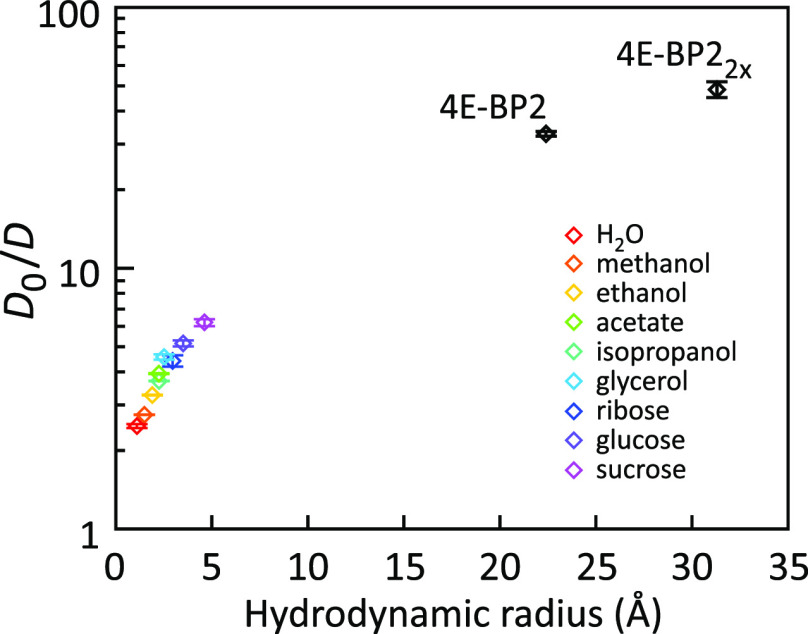
Slowing of translational diffusion of probes of different size in Ddx4 condensates relative to the buffer.^81^*D*_0_ is the translational diffusion coefficient in the buffer and *D* the diffusion coefficient in the Ddx4 condensate. The *D*_0_/*D* ratio increases with probe size, indicating a length scale dependency of condensate viscosity. Adapted with permission from ref (81). Copyright 2017 National Academy of Sciences under CC-BY license.

Taking into account these results obtained for IDPs in crowded conditions, it is likely that in IDP condensates different motions, such as translational diffusion and local and segmental chain dynamics, are impacted to different extents. Indeed, a length-scale dependence of viscosity was observed in the condensates formed by the IDP chain of Ddx4.^81^ Inside a condensate, the Ddx4 IDP chain retained fast picosecond-to nanosecond time scale dynamics despite a drastically slower translational diffusion (section 2.2.3.3).^81^ To explain this effect, the translational diffusion coefficients of a number of small probe molecules and proteins dissolved in the condensate were measured using pulse field gradient NMR diffusion spectroscopy. Indeed, the diffusion coefficients decreased monotonically with increasing hydrodynamic radius of the probe (Figure 11),^81^ as predicted by the length-scale model.

#### 2.2.3 Picosecond-to-Nanosecond Dynamics in IDP Condensates at the Atomic Level

##### 2.2.3.1 Fast IDP Motions Inside Condensates

**Figure 12:**
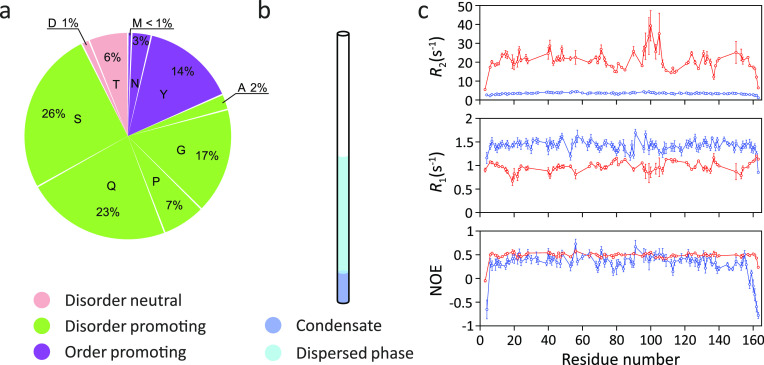
Changes in ^15^N spin relaxation rates in the FUS LC domain upon LLPS. (a) Amino acid composition of the FUS LC domain. Residues were categorized by their structural tendencies according to ref (173). (b) Cartoon representation of the condensate of the FUS LC domain in an NMR tube. (c) Residue-specific *R*_2_ and *R*_1_ rates as well as ^15^N–^1^H heteronuclear NOE values in the phase-separated (red) and dispersed (blue) state at 25 °C, 19.98 T.^83^ LLPS-induced changes in the ^15^N spin relaxation rates are consistent with slower local backbone sampling and chain dynamics inside the condensate. Figure 12c was adapted with permission from ref (83). Copyright 2015 Elsevier Inc.

While still in its infancy, first studies have started to investigate residue-specific dynamics of IDPs inside condensates. In the LC domain of FUS, the ^15^N transverse relaxation rate *R*_2_ strongly increased upon LLPS from <5 s^–1^ to 15–35 s^–1^ (Figure 12).^83^ Notably, ^15^N *R*_2_ relaxation in unfolded proteins and IDPs has been linked to segmental chain dynamics.^127^ It is therefore likely that the LLPS-induced increase in the ^15^N transverse relaxation rate *R*_2_ is caused by the slowing down of segmental dynamics. In addition, the ^15^N–^1^H heteronuclear NOEs slightly increased in the FUS LC domain upon LLPS (Figure 12). Because ^15^N–^1^H heteronuclear NOEs are more sensitive to high frequency motions, the data indicate that concentrating the FUS LC domain into condensates only slightly restricts or slows high-frequency motions but predominantly slows down segmental chain dynamics.

**Figure 13:**
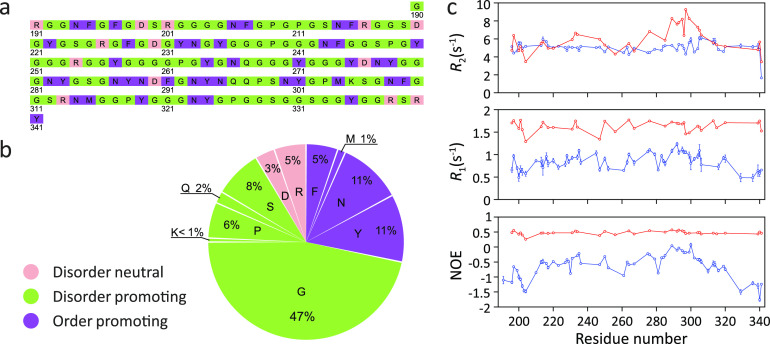
Changes in ^15^N spin relaxation rates in the LC domain of hnRNPA2 upon LLPS. (a,b) Amino acid composition of the LC domain of hnRNPA2. Residue structural tendencies reported as in ref (173). (c) Residue-specific *R*_2_ and *R*_1_ rates as well as ^15^N–^1^H heteronuclear NOE values in the LC domain of hnRNPA2 in the phase-separated (red) and dispersed (blue) states at 65 °C, 19.98 T.^84^ Measured values are consistent with slower local backbone sampling in the condensate. However, the *R*_2_ relaxation rate values, which were measured at 65 °C,^84^ are likely influenced by the fast amide hydrogen exchange at this high temperature, i.e., they do not exclusively report on IDP dynamics.^84^ Figure 13c was adapted with permission from ref (84). Copyright 2018 Elsevier.

In the LC domain of the hnRNPA2 protein, a component of RNA-processing membraneless organelles,^174,175^^15^N *R*_1_ rates as well as ^15^N–^1^H heteronuclear NOEs increase upon LLPS (Figure 13).^84^ The increased ^15^N–^1^H heteronuclear NOEs pointed to slowed or restricted local motions. However, ^15^N *R*_2_ rates were quite similar before and after LLPS (Figure 13). Because the NMR relaxation measurements were performed at 65 °C, the ^15^N *R*_2_ rates might be influenced or even be dominated by fast amide hydrogen exchange with water.^84^ Further measurements at physiological temperatures are thus required to dissect the influence of LLPS on the conformational dynamics of the LC domain of hnRNPA2.

In the phase separation of ELP_3_, an elastin-like polypeptide, the translational diffusion rate decreased by 2 orders of magnitude, from ≈100 μm^2^/s to ≈1 μm^2^/s.^85^ LLPS of ELP_3_ also resulted in an increase in ^15^N–^1^H heteronuclear NOEs and ^15^N *R*_2_ relaxation rates (from ≤5 s^–1^ to >15 s^–1^ at 14.1 T, 37 °C), consistent with a slow down or restriction of both local motions and chain reconfigurations, respectively.^85^

In these pioneering NMR relaxation studies of IDP dynamics under LLPS conditions, only a reduced set of spin relaxation rates was acquired (^15^N *R*_1_, ^15^N *R*_2_, and heteronuclear NOEs), which is not enough for their analysis in terms of timescales and amplitudes of motional components. Such an analysis would, however, be necessary to quantify the exact impact of LLPS on both local and segmental chain motions and delineate the contributions to the measured rates from either slower timescales of motions or reduced local chain backbone conformational entropy resulting in decreased amplitudes of local motions.

##### 2.2.3.2 Chain Flexibility and *R*_1_ Relaxation

The ^15^N spin relaxation studies available so far demonstrate that ^15^N *R*_2_ relaxation rates and ^15^N–^1^H heteronuclear NOE values increase when IDPs are concentrated inside condensates. The picture is however less clear in the case of residue-specific *R*_1_ relaxation rates. In the LC domain of hnRNPA2, phase separation was accompanied by a strong increase in ^15^N *R*_1_ rates from 0.5 to 1.25 to 1.25–1.75 s^–1^ (Figure 13). In contrast, the ^15^N *R*_1_ rates of the FUS LC domain decreased upon LLPS by approximately a factor of 1.5 from 1.3 to 1.7 s^–1^ to 0.75–1.2 s^–1^ (Figure 12). Thus, ^15^N *R*_1_ relaxation rates respond in an opposite manner to phase separation in the two systems.

**Figure 14:**
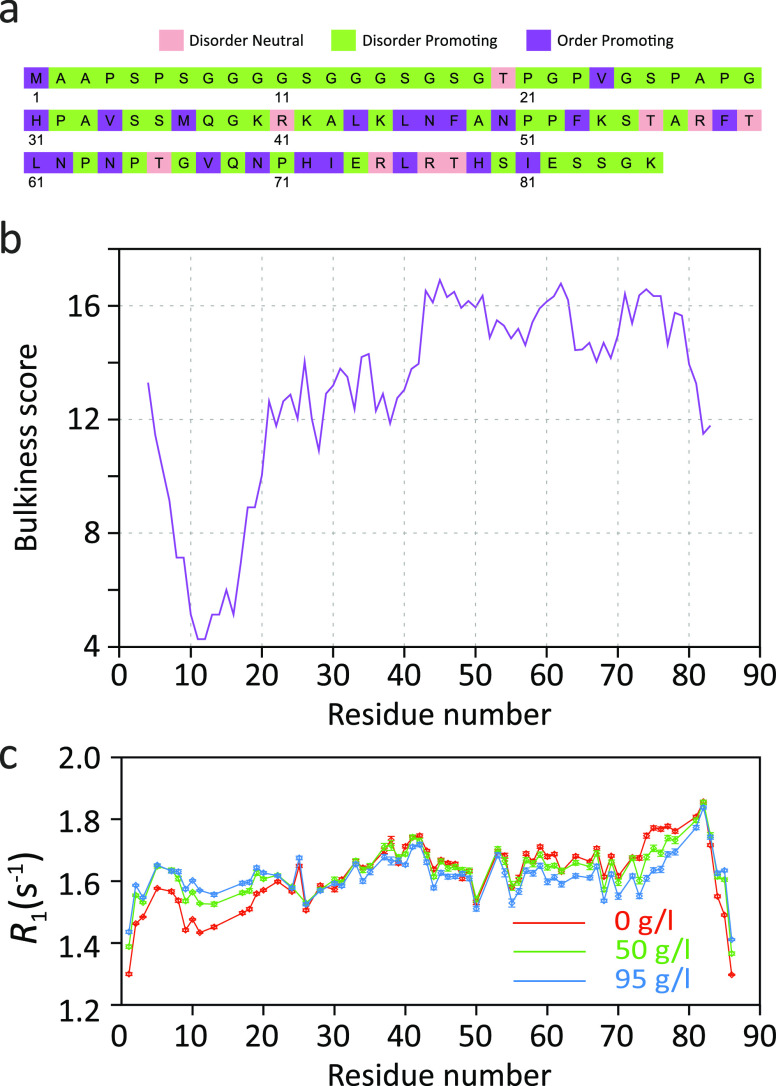
^15^N *R*_1_ relaxation in the intrinsically disordered N-terminal domain of MKK4.^172^ (a) Amino acid sequence of the N-terminal domain of MKK4. Amino acid-specific structural tendencies reported as in ref (173). (b) Amino acid bulkiness^147^ along the sequence (calculated with a window size of seven residues). (c) Residue-specific *R*_1_ relaxation rates at different concentrations of the molecular crowding agent dextran (in mg/mL).^172^ Adapted with permission from ref (172). Copyright 2019 American Chemical Society. *R*_1_ rates increase with viscosity for the highly flexible N-terminal region (first ∼25 residues) but decrease for residues 55–80.

Residue-specific differences in the sensitivity of ^15^N *R*_1_ relaxation rates to increased molecular crowding and viscosity were previously reported for the highly flexible N-terminal region of mitogen-activated kinase kinase 4 (MKK4).^172^ Addition of increasing concentrations of the molecular crowding agent dextran increased the ^15^N *R*_1_ rates for the N-terminal ∼25 residues of MKK4 (Figure 14).^172^ In contrast, lower ^15^N *R*_1_ rates were observed for residues 55–80 in the presence of high concentrations of dextran (Figure 14). Notably, neither the 25 N-terminal residues nor residues 55–80 of MKK4 contain significant amounts of regular secondary structure.^176^ Thus, even in a single disordered polypeptide chain, the ^15^N *R*_1_ relaxation rates can respond in distinct ways to increased viscosity.

**Figure 15:**
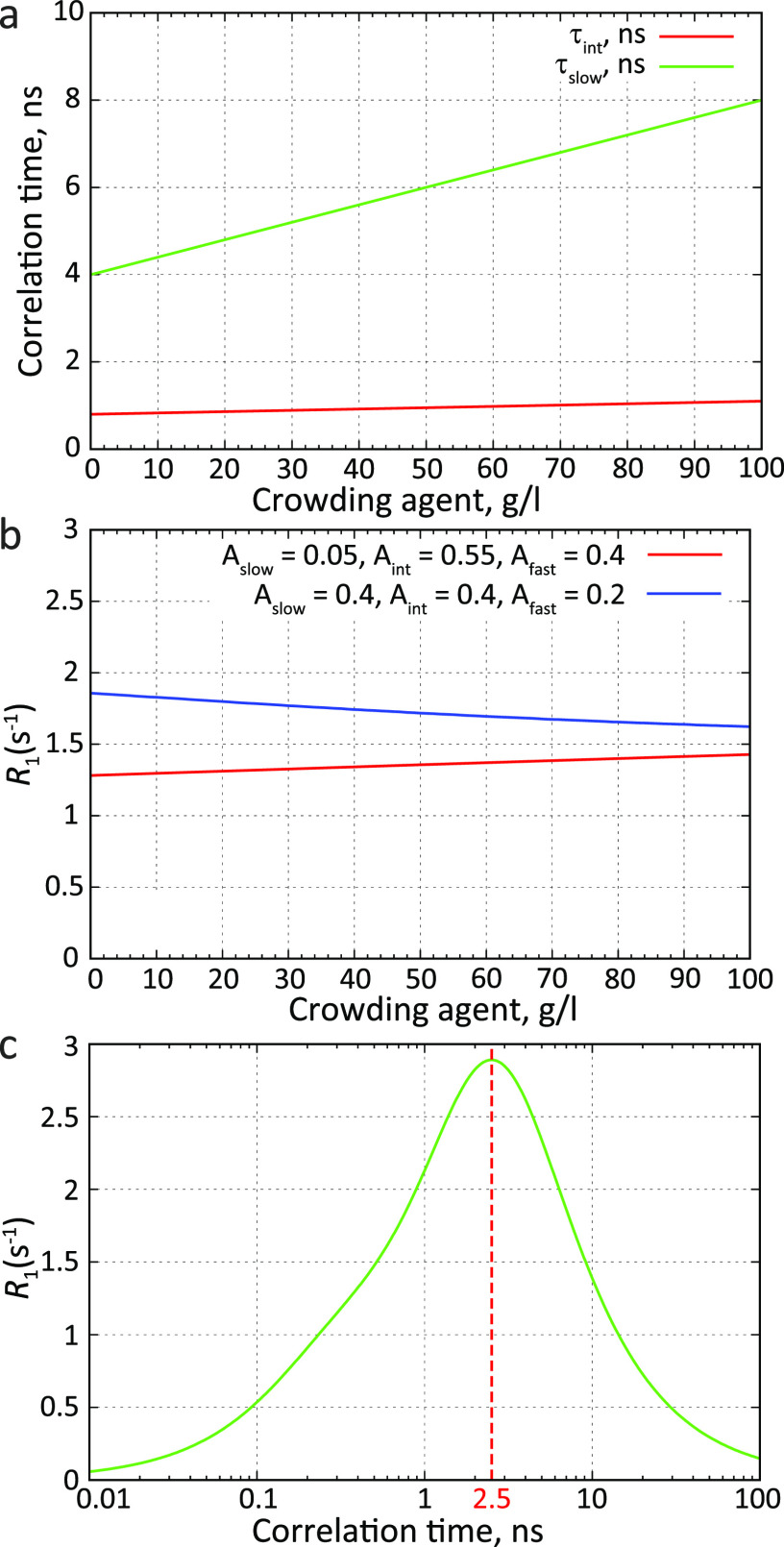
Crowding, chain flexibility, and *R*_1_ spin relaxation. (a) Dependence of motional modes on the concentration of a crowding agent. The intermediate component with the characteristic time τ_int_ is characteristic for local backbone motions. Slower chain motions are represented by the motional component with the correlation time τ_slow_. (b) *R*_1_ spin relaxation rates at different concentrations of a crowding agent for more rigid (blue) or more flexible IDP chains (red). Relaxation rates were calculated using an autocorrelation function modeled as a sum of three exponentially decaying components (Figure 4 and eqs 1 and 7 in ref (135)). (c) Dependence of the *R*_1_ spin relaxation rate on the correlation time. For fast correlation times, the *R*_1_ rate increases, reaches a maximum at ≈2.5 ns, and decreases for slower correlation times. The relaxation rate was calculated using an autocorrelation function with a single exponentially decaying component. The field was 14.1 T. The correlation time of the fastest component (τ_fast_) was set to 50 ps; the chemical shift anisotropy tensor was axially symmetric with anisotropy σ_∥_ – σ_⊥_ = −170 ppm; the N–H internuclear distance was 1.02 Å.

To gain insight into the interplay between chain flexibility and ^15^N *R*_1_ relaxation rates, we modeled the influence of molecular crowding on *R*_1_ spin relaxation rates. We took into account previous observations that increased molecular crowding predominantly impacting the characteristic times of different motional contributions but not their amplitudes.^172^^15^N *R*_1_ relaxation rates were calculated using the three-component description of the autocorrelation function (Figure 4). We used typical values of characteristic times and amplitudes obtained from ^15^N relaxation measurements in flexible and rigid parts of MKK4 at different concentrations of the crowding agent.^172^ Molecular crowding strongly slows down slower chain motions represented by the slow component with the characteristic time τ_slow_ (Figure 15a). In addition, the intermediate component with the characteristic time τ_int_, which represents local backbone motions, slightly increased with increased viscosity (Figure 15a). The flexible parts of MKK4 had a major contribution of the intermediate component (*A*_int_ > 0.5) and a very small contribution of the slow component (*A*_slow_ < 0.1). The more rigid parts of MKK4, on the other hand, had a sizable contribution of the slow component (*A*_slow_ ≈ 0.3–0.4).

To represent both a more flexible and a more rigid IDP, we compared the two three-component models *A*_slow_ = 0.05/*A*_int_ = 0.55/*A*_fast_ = 0.4 and *A*_slow_ = 0.4/*A*_int_ = 0.4/*A*_fast_ = 0.2, respectively. For the motional model with a smaller amplitude of slow motions (*A*_slow_ = 0.05), the ^15^N *R*_1_ rate increased with increasing the concentration of the crowding agent (red line in Figure 15b). In contrast, the ^15^N *R*_1_ rate decreased from 2 s^–1^ to 1.5 s^–1^ for the slow motion model (*A*_slow_ = 0.4; blue line in Figure 15b). When a single exponentially decaying component defines the autocorrelation function, the ^15^N *R*_1_ rate behaves nonlinearly with its correlation time: for correlation times below ∼2.5 ns, *R*_1_ increases, while above ∼2.5 ns it decreases (Figure 15c). Therefore, when faster (τ_fast_, τ_int_ < 2.5 ns) and slower (τ_slow_ > 2.5 ns) components define chain dynamics simultaneously, the ^15^N *R*_1_ rate behavior depends on the contribution of each component. This analysis shows that in a flexible chain, where faster/intermediate backbone motions have a major contribution (*A*_int_) to the correlation function, *R*_1_ values will increase with slowing down of characteristic times of motions in more crowded conditions. In a more rigid chain, where these motions are more restricted and their contribution is low, *R*_1_ values will decrease with crowding. Depending on the relative contribution of different motional modes to the correlation function, *R*_1_ relaxation rates can thus decrease or increase upon LLPS of IDPs.

We also point out that ^15^N–^1^H heteronuclear NOEs in the monomeric state of FUS LC are positive (Figure 12c), consistent^177^ with more restricted backbone dynamics than in hnRNPA2, where negative NOE values are present (Figure 13c). In addition, the LC domains of hnRNPA2 and FUS differ in their compaction. Despite similar lengths of the two proteins (163 residues for FUS; 151 residues for hnRNPA2), the FUS LC domain has a larger hydrodynamic radius (3.32 nm) than the LC domain of hnRNPA2 (2.89 nm).^84^ This difference in hydrodynamic radii can be explained by the collapse of hnRNPA2 LC, which is favored by its high glycine content (47%) (Figure 13b). Both data support a higher flexibility of the hnRNPA2 backbone when compared to the polypeptide chain of the LC domain of FUS. The decrease in the ^15^N *R*_1_ relaxation rates in FUS LC upon phase separation (Figure 12c) is therefore likely a consequence of its more rigid (when compared to hnRNPA2) backbone with lower local conformational entropy.

In the analysis of LLPS behavior of ^15^N *R*_1_ relaxation rates provided here, the chain flexibility (the amplitudes of chain conformational sampling) was assumed to remain constant upon LLPS. As we will discuss later, this is not necessarily true: IDP chains can become even more rigid upon phase separation. In the case of FUS LC, this effect could decrease ^15^N *R*_1_ values even further in the condensed phase.

##### 2.2.3.3 Molecular Crowding and Intermolecular Contacts

**Figure 16:**
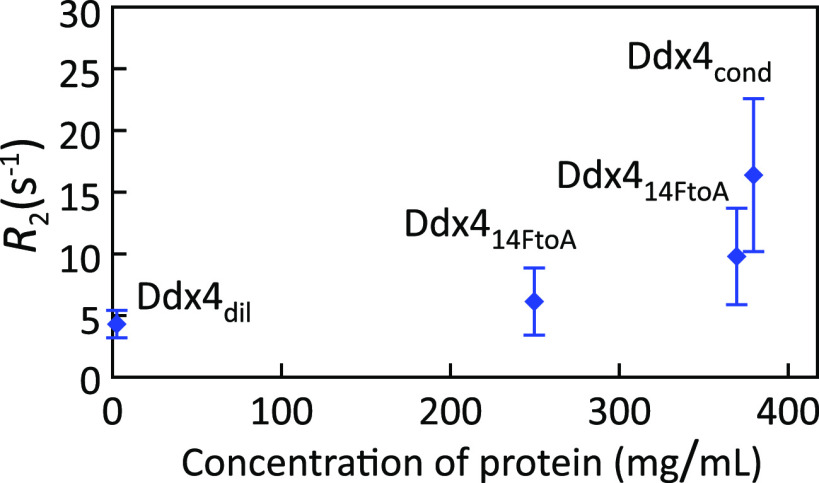
^15^N *R*_2_ relaxation rates in the intrinsically disordered N-terminal domain of Ddx4, in the dilute phase (Ddx4_dil_) and in the condensed phase (Ddx4_cond_).^81^ To mimic the high condensate viscosity, a mutant of Ddx4 (Ddx4_14FtoA_) was created, in which 14 phenylalanine residues were mutated to alanine. This mutant can reach concentrations close to those observed inside the condensate (380 mg/mL) but does not phase separate. Relaxation rates were measured in Ddx4_14FtoA_ concentrated to 250 and 370 mg/mL (concentrations determined from absorption at 280 nm). The difference in *R*_2_ values observed for Ddx4_14FtoA_ at 370 mg/mL and Ddx4_cond_ at 380 mg/mL were attributed to the influence of intermolecular contacts in Ddx4_cond_ that mediate phase separation.

LLPS-induced slowing of IDP backbone motions was also reported for the intrinsically disordered N-terminal 236 residues of the germ-granule protein Ddx4.^81^ The measured diffusion constant of Ddx4 in the condensed phase was very small, 0.75 ± 0.04 μm^2^/s. This value corresponds to a protein of an apparent hydrodynamic radius of ∼550 nm in dilute aqueous solution. Despite such slow apparent reorientational dynamics, amide resonances and sharp ^13^C side-chain resonances were present in the NMR spectra, indicating that local motions are still rapid inside the condensate. Residue-specific ^15^N *R*_2_ relaxation rates increased from 1 to 6 s^–1^ in the dilute phase to 16.4 ± 6.1 s^–1^ in the condensed phase (at 18.8 T, 30 °C)^81^ (Figure 16). On the other hand, ^15^N–^1^H heteronuclear NOEs, which are sensitive to local motions on the pico- to nanosecond time scale, did not change significantly upon LLPS, in agreement with the observed line width of the NMR signals. Brady et al. further analyzed the ^15^N *R*_1_/*R*_2_ relaxation rates and ^15^N–^1^H heteronuclear NOEs in terms of the *S*^2^τ_c_ product. *S*^2^τ_c_ is the square of the order parameter *S*, which describes the amplitude of the amide bond vector motions, multiplied by the residue-specific chain tumbling time τ_c_. The analysis yielded *S*^2^τ_c_ = 1.3 ± 0.6 ns for the dilute phase of Ddx4, and 8.5 ± 3.0 ns for the condensed phase. The dynamics of the disordered Ddx4 chain are thus slower inside the condensate.

Slowed conformational dynamics of IDPs can arise from both increased viscosity and intermolecular contacts inside condensates. To evaluate to which extent higher *R*_2_ relaxation rates and *S*^2^τ_c_ values in the condensate can exclusively be attributed to increased molecular crowding, a mutant of Ddx4 was constructed in which all 14 phenylalanines were mutated to alanine (termed Ddx4_14FtoA_; Figure 16). Ddx4_14FtoA_ can be concentrated to 370 mg/mL without undergoing phase separation. The concentration of 370 mg/mL of Ddx4_14FtoA_ in the nonphase-separated state is similar to the concentration of the wild-type protein in the condensed phase (380 mg/mL). ^15^N *R*_2_ relaxation rates measured in Ddx4_14FtoA_ were 9.8 ± 3.9 s^–1^, and the *S*^2^τ_c_ product was ∼5.3 ns. Despite similar levels of molecular crowding, both the ^15^N *R*_2_ relaxation rates and the *S*^2^τ_c_ product was lower for the Ddx4_14FtoA_ mutant protein when compared to wild-type Ddx4 inside the condensate (^15^N *R*_2_ = 16.4 ± 6.1 s^–1^; *S*^2^τ_c_ = 8.5 ± 3.0 ns). The additional slowing of chain dynamics in the condensed phase compared to the Ddx4_14FtoA_ mutant was attributed to intermolecular contacts. At the same time, the removal of 14 aromatic phenylalanine side chains in Ddx4_14FtoA_ might have had itself an impact on the chain flexibility and thus on the ^15^N *R*_2_ relaxation rates, for example, via the reduction of the number of intramolecular hydrophobic interactions.

**Figure 17:**
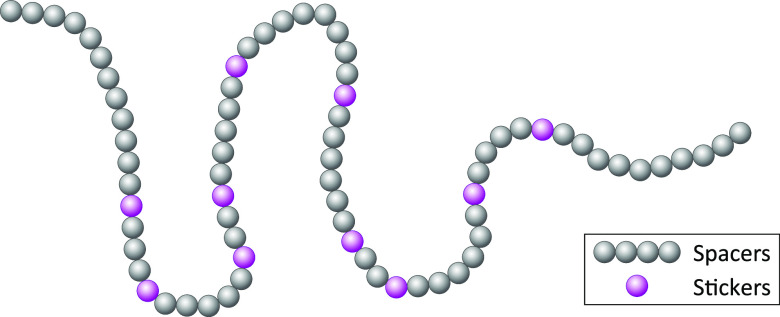
Cartoon representation of a protein chain illustrating the stickers-and-spacers model.^178^ Some residues or groups of residues, which are often aromatic or contain aromatic residues and facilitate cross-linking between different protein molecules in condensates, act as “stickers”. Residues between “stickers” are called “spacers”, which modulate the formation of the contacts between “stickers”.

Biomolecules that phase separate often contain multiple sites that engage in intra- and intermolecular interactions, a feature that is called multivalence. Multivalent molecules have a tendency to assemble into larger complexes, which increases with the number (valence) and affinity of the interacting sites. This assembly decreases their solubility and promotes phase separation, which in turn facilitates intermolecular contacts and promotes the formation of even larger complexes.^10^ The two processes are coupled, resulting in the formation of interconnected networks of macromolecules and a density increase in the condensed phase.^178^ Mittag and co-workers adapted a stickers-and-spacers model, developed for associative polymers,^179,180^ to simulate the phase separation behavior of intrinsically disordered prion-like domains (PLDs) and LC domains.^178^ They then applied the analysis to the phase separation of the LC domain of heterogeneous nuclear ribonucleoprotein A1 (hnRNPA1 LC). Residues or groups of residues that are involved in intermolecular interactions promoting phase separation were called “stickers”, and other residues that are interspersed between stickers were called “spacers” (Figure 17). “Stickers” were associated with aromatic residues, which are often present in PLDs and contribute to their phase separation.^41,98,181^ This assignment was substantiated by experimentally measured ^15^N *R*_2_ NMR spin relaxation rates, featuring elevated *R*_2_ rates around aromatic residues, reporting on restricted chain motions. A lattice-based coarse grained model with a single bead (sticker or spacer) representing each residue was used to simulate phase separation binodals. In the model, sticker–sticker interactions were set to be more energetically favorable than sticker–spacer and spacer–spacer interactions. Simulated binodals for wild-type hnRNPA1 LC and several mutants (having more or less aromatic residues than the wild-type protein) matched experimentally determined phase transition points. The critical temperature and the width of the two-phase regime increased with an increasing number of aromatic residues in hnRNPA1 LC variants, emphasizing the role of valence in phase separation.

Notably, intermolecular interactions that promote LLPS are not only restricted to interactions between aromatic residues.^10,182^ Electrostatic interactions between segments or residues of opposite charge^98,99,183−185^ were also found to drive LLPS. In addition, noncharged residues can participate in LLPS. Polyglutamine polymers were found to phase separate, possibly through the formation of hydrogen bonds.^186^ In elastin-like polypeptides, contacts between Ala, Val, and Pro residues were formed in the condensate, suggesting the role of intermolecular hydrophobic interactions in LLPS.^85^ Kim et al. combined a specific labeling scheme with edited-filtered nuclear Overhauser effect spectroscopy (NOESY) to probe intermolecular contacts relevant for the LLPS of the C-terminal intrinsically disordered region of RNA-binding CAPRIN1 protein at a residue-specific level.^182^ Whereas the importance of the interaction between aromatic side chains for CAPRIN1 LLPS was confirmed, a previously not fully appreciated role of backbone interactions, in particular H^α^–H^N^ contacts involving aromatic and nonaromatic residues, was established. These contacts were suggested to be stabilized through amide hydrogen bond formation and/or π–π interactions. Indeed, three short regions with significantly increased aliphatic and aromatic to amide proton NOEs in CAPRIN1 condensate, ^624^GYR^626^, ^638^GYR^640^, and ^660^RDYSGYQ^666^, were identified. Mutating the first three residues in these regions to ASA attenuated LLPS. NMR ^15^N *R*_2_ spin relaxation rates were also particularly elevated in the ^638^GYR^640^ and ^660^RDYSGYQ^666^ regions both in monomeric CAPRIN1 and in the CAPRIN1 condensate, indicating the presence of intramolecular interactions restricting its chain dynamics. Notably, *R*_2_ relaxation rates decreased significantly in these regions upon the LLPS-disrupting mutation to ASA, suggesting a link between intramolecular interactions in the monomeric CAPRIN1 and the intermolecular interactions stabilizing LLPS.

#### 2.2.4 IDP Dynamics Probed by Reporters

**Figure 18:**
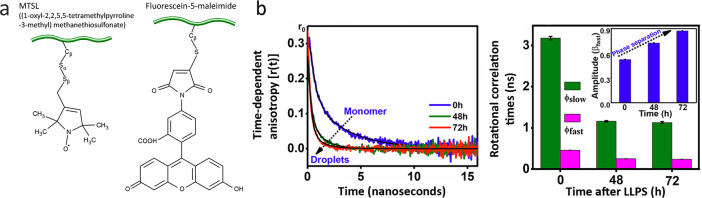
IDP dynamics probed by fluorescence anisotropy and electron paramagnetic resonance (EPR) spectroscopy. (a) Cartoon representation of a MTSL nitroxide spin label used in EPR spectroscopy (left) and of a fluorescein fluorophore (right) used in fluorescence anisotropy measurements. Both methods measure the dynamics of the probe covalently attached to an IDP chain, and its own rotational mobility relative to IDP backbone may contribute to the apparent dynamic parameters of the IDP chain. (b) Fluorescence anisotropy decay curves in the solution of monomeric human tau K18 (0 h) and in droplets 48 h and 72 h after phase separation (left panel).^87^ Correlation times of slow and fast components and the amplitude of the fast component obtained by a biexponential fit of decay curves (right panel). Reprinted with permission from the *Journal of Physical Chemistry Letters*, Volume *10*, Issue 14, Majumdar, A.; Dogra, P.; Maity, S.; Mukhopadhyay, S. Liquid–Liquid Phase Separation Is Driven by Large-Scale Conformational Unwinding and Fluctuations of Intrinsically Disordered Protein Molecules, pages 3929–3936 (ref (87)). Copyright 2019 American Chemical Society.

Conformational dynamics of IDPs inside condensates can be investigated by time-resolved fluorescence anisotropy decay and continuous-wave electron paramagnetic resonance (EPR). Both methods provide information on the correlation function of the probe on the picosecond-to-nanosecond time scale. In the case of time-resolved fluorescence anisotropy decay, the probe is a fluorescent label that is covalently attached to one of the residues of the IDP. For EPR, it is a spin label. Both methods thus probe chain dynamics indirectly through the dynamics of the attached label (Figure 18a). However, in contrast to the NMR relaxation studies of IDP dynamics, correlation functions obtained in fluorescence anisotropy measurements can directly provide some quantitative information about amplitudes and time scales of IDP motions on the range of timescales around the lifetime of the fluorophore (several nanoseconds), albeit not at a residue-specific level.

##### 2.2.4.1 Fluorescence Anisotropy

Fluorescence anisotropy was used to probe LLPS-induced changes in the conformational dynamics of the intrinsically disordered protein α-synuclein.^59^ Experimental fluorescence anisotropy decay curves were fitted in the dispersed monomeric state and the condensed phase with a single exponential decay. From this analysis, rotational correlation times of 1.0 and 1.6 ns were derived for α-synuclein outside and inside the droplets, respectively. In addition, Ray et al. noted that the decay curve had a positive *y*-intercept (∼0.1), consistent with the appearance of the contribution of much slower (>10 ns) motions inside droplets.^59^ The amplitude of faster motions is therefore restricted inside droplets, consistent with reduced chain flexibility and decreased local conformational entropy. This explanation was also provided by the authors,^59^ who related the chain rigidification to the specific intermolecular interactions that drive α-synuclein droplet formation.

Increased rotational correlation times were also observed upon phase separation of the full-length human tau protein htau40.^187^ To this end, four double-cysteine mutants, Tau_17/244_, Tau_149/244_, Tau_244/354_, and Tau_354/433_ (position of first cysteine mutation/position of second cysteine mutation), were prepared and labeled them the fluorescent dye AF488. In the monomeric form of htau40, the fluorescence anisotropy curves were best fitted with correlation times of 1.9 ± 0.2 ns, 1.5 ± 0.1 ns, 1.5 ± 0.1 ns, and 1.2 ± 0.1 ns for Tau_17/244_, Tau_149/244_, Tau_244/354_, and Tau_354/433_, respectively. In droplets, these correlation times increased to 4.7 ± 0.7 ns, 2.6 ± 0.2 ns, 3.2 ± 0.2 ns, and 2.7 ± 0.2 ns, respectively. However, in contrast to the α-synuclein study, no contribution of slower motions appeared in fluorescence anisotropy decay curves in droplets.

Fluorescence anisotropy decay in IDPs is also often analyzed with multiple exponential decay components.^188,189^ To probe the conformational dynamics of the repeat domain of the intrinsically disordered protein tau in liquidlike droplets, the fragment tau-K18 (residues 244–372 of htau40) was labeled at the two native cysteine residues Cys291 and Cys322. To describe the rotational tumbling of the fluorescent probe, the observed time-resolved fluorescence anisotropy decay was fitted with a sum of two exponential components.^87^ The correlation time of the slow component decreased from ∼3 ns to ∼1.1 ns upon LLPS. In parallel, the correlation time of the fast component decreased from 440 to 240 ps, whereas its contribution increased from 52% to 73% (Figure 18b). This would point to faster dynamics in tau-K18 inside droplets, whereas the inverse effect, i.e., deceleration of the conformational dynamics in IDPs upon LLPS, is generally observed. According to this model, the tau chain in the monomeric state is partially collapsed and the slow component represents its global tumbling. Upon LLPS, the tau chain would undergo a conformational expansion, and the slow component now represents the backbone torsional mobility in the dihedral angle space of expanded disordered conformations, potentially explaining its faster correlation time.^87^ A similar behavior was observed by Dogra et al. using fluorescence anisotropy in the LLPS of the disordered oligopeptide repeat domain of the melanosomal protein Pmel17.^190^ The fluorescence anisotropy decay curves were fitted with two components. The correlation time of the slow component decreased from 1.76 to 1.1 ns upon LLPS, and the correlation time of the fast component decreased from 390 to 230 ps.

The observed changes in the fluorescent anisotropy decay of tau-K18 and Pmel17 point to different conformational dynamics of the proteins before and after LLPS. However, it is less clear if these changes can be connected to changes in the overall compaction of these IDPs upon phase separation. One limitation for example is related to the description of the dynamics of IDPs in terms of global tumbling. Excluding the cases where the IDP chain is largely collapsed, NMR spin relaxation^68,127,128^ and fluorescence anisotropy^129^ studies questioned the appropriateness of describing the dynamics of IDPs in terms of overall or global tumbling. Instead, a notion of motions of chain segments or segmental motions was proposed. The ∼3 ns correlation time of the slow component, which was observed for nonphase separated tau-K18 at 25 °C, is in the 2–6 ns range of the slow component characteristic time as detected by NMR spin relaxation in an IDP chain without secondary structure elements at 25 °C.^135^ On the other hand, the value of ∼1.1 ns obtained upon LLPS is more close to the range of 0.5 to 1.3 ns, which was assigned to local backbone sampling motions.^135^ It is therefore possible that in tau-K18, the long-range chain motions actually become much slower upon LLPS, as reported in other systems, but its contribution to the decay curve is small. In addition, the apparent decrease in both the slow and fast motional components upon LLPS might result from an averaging between the time scales of chain dynamics, local backbone sampling, and rotational mobility of the fluorescent probe (Figure 18a). In NMR spin relaxation studies, when two components were used instead of three to describe IDP dynamics, their characteristic times were situated in between the characteristic times obtained by a three-component analysis.^134^

##### 2.2.4.2 EPR Spectroscopy

**Figure 19:**
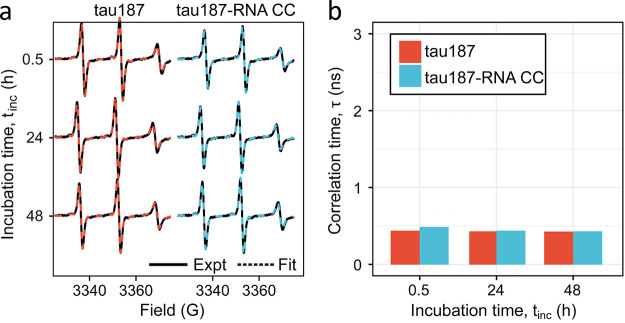
Dynamics in the tau fragment Δtau187 probed by continuous-wave EPR spectroscopy.^191^ (a) X-band continuous wave EPR spectra measured in monomeric Δtau187 solution and in the condensate formed by complex coacervation of Δtau187 with RNA (tau187-RNA CC) at different times of incubation at room temperature. Measured EPR profiles (solid line) were fitted with single-component simulation (dashed line). (b) Rotational correlation times obtained from EPR simulations. Adapted from ref (191) under the terms of the CC-BY 4.0 license.

LLPS-induced changes in the conformational dynamics of a fragment of tau were probed by continuous-wave EPR spectroscopy.^45^ To this end, the tau fragment Δtau187 (residues 225–441 of htau40) was spin-labeled at cysteine 322. Subsequently, Δtau187 was concentrated into droplets through complex coacervation with RNA. Continuous-wave EPR spectra were recorded at 25 °C. No change in the EPR profile was observed upon complex coacervation (Figure 19). The overlapping EPR profiles were fitted with a single rotational correlation time of 425 ± 16 ps. Lin et al. therefore suggested that Δtau187 inside of RNA-induced droplets has the same dynamical properties as in the monomeric state.^45^

Because different IDPs and IDP fragments might behave differently upon LLPS, in particular when LLPS is induced in different ways (for example, self-coacervation versus complex coacervation), Δtau187 in droplets might indeed retain the same dynamical properties as in the monomeric state. At the same time, it is important to note that EPR spin labels and fluorescence tags attached to an IDP chain may have intrinsically larger amplitudes of reorientation relative to the IDP backbone. The time scale of 425 ps obtained by Lin et al. in Δtau187 thus might result from the averaging of time scales of Δtau187 local backbone dynamics and the rotational mobility of the spin label (Figure 18a). Fast motions of the probe (spin label or fluorescent tag) may dominate the autocorrelation function that describes the reorientation rendering EPR spectroscopy and fluorescence anisotropy experiments less sensitive to the slower motions of the IDP backbone chain.

#### 2.2.5 Microsecond-to-Millisecond Exchange Processes

Phase separation of multiple IDPs is followed by the maturation of liquid droplets and, ultimately, protein aggregation.^105−107^ The latter may be facilitated by the presence of transiently populated conformations with secondary structure elements. For example, intrinsically disordered tau protein conformations with β-structure propensity were linked to its pathologic aggregation.^192^ At the same time, multivalence-driven formation of IDP oligomers was suggested to favor the phase-separation itself.^10^ Therefore, the characterization of conformational states of IDPs that favor intermolecular contacts would be important to understand the mechanisms of LLPS and aggregation.

**Figure 20:**
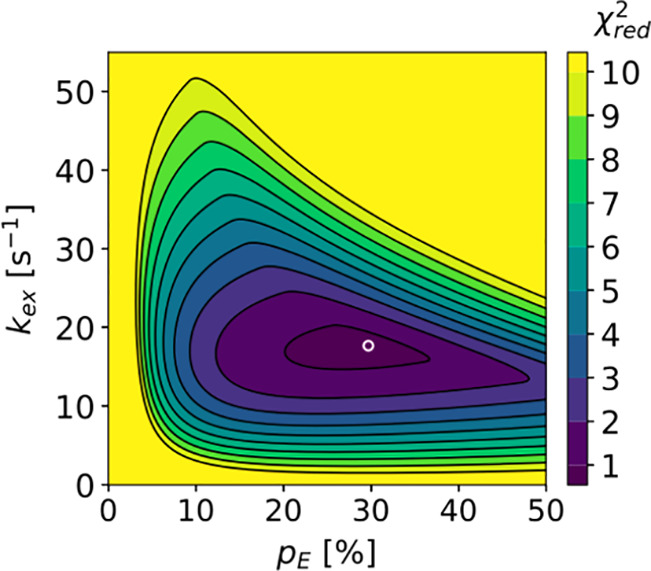
Conformational exchange between the ground state and an excited state of Ddx4 in a condensate. χ^2^ values of *p*_E_ (excited state population) and *k*_ex_ (exchange rate) fit of *R*_1ρ_ relaxation dispersion profiles.^89^ Optimal values (*p*_E_ = 29.7%, *k*_ex_ = 17.7 s^–1^) are indicated by the white circle. Reprinted with permission from the *Journal of the American Chemical Society*, Volume *140*, Issue 6, Yuwen, T.; Brady, J. P.; Kay, L. E. Probing Conformational Exchange in Weakly Interacting, Slowly Exchanging Protein Systems via Off-Resonance *R*_1ρ_ Experiments: Application to Studies of Protein Phase Separation, pages 2115–2126 (ref (89)). Copyright 2018 American Chemical Society.

^15^N *R*_2_ and *R*_1ρ_ spin relaxation dispersion might be particularly useful to characterize microsecond-to-millisecond exchange processes and the associated conformational states.^193−195^ Yuwen et al. used off-resonance ^15^N *R*_1ρ_ relaxation dispersion to probe conformational exchange in the condensed phase of the intrinsically disordered domain of Ddx4.^89^ Ddx4 was found to undergo exchange on a millisecond time scale (*k*_ex_ = 17.7 s^–1^) between a ground state and a significantly populated (∼30%) excited state (Figure 20). The excited state was characterized by elevated ^15^N *R*_2_ relaxation rates (*R*_2,excited_ = 4.5 ± 2.7 × *R*_2,ground_). The elevation in ^15^N *R*_2_ rates was suggested to arise from enhanced intermolecular contacts in the excited state.^193^ However, a similar exchange process was observed for the nonseparating Ddx4_14FtoA_ mutant at a concentration mimicking that of the condensed phase (370 mg/mL), questioning the specificity of the exchange process for the condensed phase. Further work will therefore be required to elucidate the importance of microsecond-to-millisecond exchange processes in the structural and dynamical properties of IDPs inside condensates.

Single-molecule fluorescence-based methods can also be employed to detect different conformational populations of IDPs. Manger et al. reported the presence of two conformations of full-length tau protein (htau40), stable on time scales of multiple seconds, based on a difference in their steady-state fluorescence anisotropies.^189^ htau40 has two naturally occurring cysteines, C291 and C322, which were mutated to serine, and tyrosine Y310 was instead mutated to a cysteine, to which the fluorescent ATTO647N dye was attached. To be able to collect enough photons for statistics and discern multiple populations, htau40 molecules had to be observed for several seconds without perturbative immobilization. For this purpose, Manger et al. trapped them inside an anti-Brownian electrokinetic trap. Two conformational states of htau40 had anisotropy distributions peaked at 0.17 (lower anisotropy peak) and 0.21 (upper anisotropy peak). When the molecules were trapped in the electrokinetic trap for over an hour, the lower anisotropy peak became favored over the upper, indicating conformational conversion over a time scale of tens of minutes. To understand the nature of the difference between these two states, Manger et al. performed time-resolved fluorescence anisotropy measurements of the ensemble of htau40 molecules and fitted anisotropy decay curves with three components, the fastest component (φ_f_) being assigned to dye rotations around the flexible linker, the intermediate component (φ_i_) to protein segmental motions, and the slower component (φ_s_) to longer segmental motions or a global motion around the shorter axis of the IDP. Component correlation times were φ_f_ = 0.25 ns, φ_i_ = 5.3 ns, and φ_f_ = 118 ns, and their amplitudes, expressed in terms of cone half angle, were θ_f_ = 29° and θ_i_ = 30°. The obtained values were partitioned into two conformation-specific values to explain the observed partition of steady-state anisotropies. Partition of correlation time values resulted in highly spread numbers (φ_i_ = 1.1 ns, 10.5 ns; φ_s_ = 11.3 ns, >1 μs; for any φ_f_ value, the lower anisotropy peak 0.17 could not be obtained), suggesting that the motional amplitudes should be different. Based on these results, it was suggested that one of the htau40 conformations should be more compact than the other. Because no conformational populations of htau40 were observed in solution previously by FRET measurements,^196^ Manger et al. concluded that the differences in structure should be fairly subtle. We note in addition that it is also possible that one htau40 conformation is not significantly more compact than the other but rather has more restricted backbone dynamics, explaining lower motional amplitudes of fast and/or intermediate components.

#### 2.2.6 Molecular Dynamics Simulations of IDPs in Condensates

Different conformational states of IDPs can possess distinct dynamic properties. Most experimental methods, however, offer only an ensemble-averaged view of IDP dynamics. More detailed insights into the dynamics of each conformational state of an IDP can potentially be offered by computational methods, such as molecular dynamics (MD) simulations.^197^ In addition, the simulations may help us in elucidating the nature of intermolecular contacts that drive and stabilize LLPS in different systems. However, MD simulations of molecular condensates are very challenging due to a very high molecular density and, consequently, a very high number of atoms in the simulation. A detailed review of recent advances in this field is given by Shea et al.^198^ Currently there are two approaches to carry out MD simulations in condensates: either include all atoms in the molecule into the simulation (all-atom simulations) or represent the protein molecule as a sequence of beads, with the bead number ranging from one per molecule to several per residue (coarse-grained simulations).^198^

All-atom MD simulations could in theory be used to predict chain motions occurring in IDPs on timescales that are significantly faster than the trajectory length. For a typical MD trajectory of several microseconds, that would mean sampling conformational dynamics on timescales up to hundreds of nanoseconds. However, the obtained conformational states and their dynamic parameters can only be considered accurate when the conformational sampling inside trajectories agrees with the experimentally derived data, such as small-angle X-ray scattering, NMR, and fluorescence spectroscopy parameters. For IDPs, unfortunately, that is still not entirely the case, because the force fields and water models used in MD simulations were optimized mostly for folded proteins.^199^ Rauscher et al. reported that conformational ensembles generated using different MD force fields differ significantly from each other and do not match the experimental data.^200^ In particular, IDPs in MD simulations tend to be more compact than determined by experimental measurements^201,202^ and oversampled helical conformations.^203^ Inaccuracies in the solvent–solvent and solvent–protein interactions^201,204,205^ that result in a poor solvation of IDPs were targeted to improve water models and force fields. Polarizable force fields were also proposed to better simulate water–protein interactions.^206−208^ Additional improvements concerning force field backbone torsional angle sampling^203,209−212^ were proposed, including an introduction of a specific sampling for residues enriched in disordered regions.^213−217^ However, despite a remarkable progress achieved in the development of force fields and water models improving the quality of reproduction of experimental observables,^198^ there are still disagreements in particular between the helical propensity of IDPs calculated from an MD trajectory and derived from NMR data, requiring a residue-specific fine-tuning of force field parameters.^218^ Poor conformational sampling inside MD trajectories will, in its turn, inevitably result in discrepancies between experimentally measured and simulated parameters describing IDP dynamics, such as NMR relaxation rates.^219^

The application of all-atom simulations to the LLPS of IDPs is still limited by its huge computational cost and typically requires high-performance hardware.^220^ Paloni et al. performed explicit-solvent simulations of three 12-residue fragments of the N-terminal disordered region of Ddx4 protein at high concentration (∼150 mg/mL) and found intra- and intermolecular contact propensities similar to those observed experimentally.^221^ Decreased contact formation was observed in the simulations of mutants where either phenylalanines were replaced with alanines or arginines with lysines, in agreement with experimental data indicating decreased LLPS propensity for these mutants. Based on the results, it was concluded that the assembly of Ddx4 may be stabilized by clusters of arginine and aromatic residues. To reduce the computational cost, one can make use of implicit solvent; however, the match between MD trajectory conformational sampling and experimental data would be further decreased.^198,222^ One of the force field models with an implicit solvent used to reproduce IDP conformational sampling^146,223,224^ is the ABSINTH model (self-assembly of biomolecules studied by an implicit, novel, and tunable Hamiltonian).^225,226^

Coarse-grained models, on the other hand, are much more affordable computationally and were used to simulate LLPS phase diagrams of IDPs. These models are undergoing current developments that aim at improving their generality, i.e., the ability of predicting LLPS phase diagrams of a broad range of IDPs.^227,228^ Very recently, Tesei et al. developed a coarse-grained model with residue-level specificity able to predict phase separating behavior of multiple IDPs.^229^ To achieve this goal, Tesei et al. started with the hydrophobicity scale model, in which residue–residue interactions are determined by salt-screened charge–charge interactions, steric repulsion, and hydropathy, and subjected the latter parameter to multiple rounds of optimization. First, hydropathy parameters were re-evaluated based on 87 hydrophobicity scales. Subsequently, these parameters were further trained using Bayesian learning on a set of experimental SAXS and NMR paramagnetic relaxation enhancement data from 45 IDPs and data simulated from Langevin dynamic simulations with these parameters. The third validation round involved comparing intermolecular contacts predicted from two-chain simulations and experimental NMR PRE data for two IDPs, the low-complexity domains of hnRNPA2 and FUS. The obtained models succeeded quite well in reproducing the LLPS behavior of hnRNPA2 LCD, FUS LCD, multiple variants of hnRNPA1 LCD, and the disordered N-terminal region of Ddx4. In addition, the obtained models corroborated the previously proposed role of tyrosine and arginine residues as stickers driving LLPS. Finally, the optimized models were in agreement with the experimentally observed coupling between chain compaction and phase separation, and with the breakup of this coupling due to charge effects.

Most coarse-grained models used in LLPS studies are not, however, yet well suited to reproduce partial secondary structure propensity of IDPs^198,222^ and hence their conformational sampling. However, a parallel direction in the development of new coarse-grained models is aimed at improving the poor conformational sampling of IDPs present in all-atom simulations.^230−232^ Ramis et al. used a modified SIRAH coarse-grained field to study the conformational sampling of the intrinsically disordered protein α-synuclein. The simulations reproduced well the overall secondary structure content (0.2 ± 0.4% α-helix, 26.8 ± 6.8% β-sheet, and 73.0 ± 6.8% random coil) observed experimentally in circular dichroism studies (<2% α-helix, 30% β-sheet, and 68% random coil). In addition, the simulated chemical shifts (C_α_, C, N, and H_α_) showed good agreement with experimental values. However, only a weak correlation between computed and experimentally determined secondary C_α_ and C_β_ chemical shifts (difference between a chemical shift and its random-coil value for a given residue) was observed, demonstrating that the simulation was still not capable to accurately reproduce conformational sampling.

**Figure 21:**
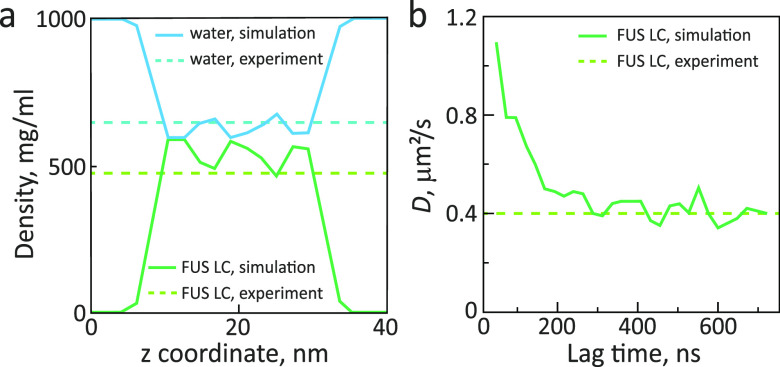
Molecular dynamics simulations of the phase-separated FUS LC.^233^ Adapted with permission from ref (233). Copyright 2020 American Chemical Society. (a) Simulated (in all-atom simulation) and experimentally determined^82^ density profiles of FUS LC. (b) Simulated (in all-atom simulation) protein self-diffusion coefficient of FUS LC in the slab along the *z*-axis as a function of the lag time. The dashed line indicates the experimentally determined^83^ value.

One of the strategies to reduce computational cost while retaining precision of all-atom simulations is to combine all-atom and coarse-grained simulations. For example, a coarse-grained simulation can be used to generate an initial equilibrated configuration of a phase-separated IDP, and the subsequent all-atom simulation will then start from the chain coordinates obtained in coarse-grained simulation. Zheng et al. used this approach to develop a more detailed picture of the intermolecular contacts that stabilize phase separation of two IDPs, FUS LC and the disordered N-terminal RGG domain of LAF-1 (LAF-1 RGG).^233^ 40 chains of the proteins were equilibrated in a planar slab geometry using coarse-grained simulations. Subsequently, a 2-μs trajectory was run with the amber ff03ws force field and TIP4*P*/2005 explicit solvent model. Good agreement between simulated and experimentally determined average density, water content, intermolecular contacts, and protein diffusivity was found (Figure 21). In both proteins, the critical role of contacts involving tyrosine and arginine residues in LLPS was supported by the MD simulation. In the case of LAF-1 RGG, which contains a significant number of charged residues, the additional role of contacts between residues of opposite charge (such as arginine and aspartate) was highlighted. In FUS LC, all intermolecular contacts except those involving tyrosine residues are primarily stabilized by hydrogen bonds, and contacts with tyrosine residues are primarily stabilized by sp^2^/π-group interactions. In LAF-1 RGG, salt bridges and cation−π interactions were found to play a significant role.

In summary, both all-atom and coarse-grained simulations could potentially be used in the future to study the dynamic behavior of IDPs inside condensates. For all-atom simulations, the main questions are the computational cost, which can only be resolved by improvements in the computer hardware and better water models and force fields to improve the accuracy of conformational sampling. For the coarse-grained simulations, the goal would be to combine improvements that aim at reproducing intermolecular contacts, which drive LLPS, and the modifications that improve IDP conformational sampling and thus dynamics.

## 3 Discussion and Perspectives

IDPs and intrinsically disordered protein regions are key components of many membraneless compartments. The dynamics in IDPs occur on multiple time and length scales: the nucleation of short α-helices occurs on timescales between picoseconds and nanoseconds,^234−236^ longer helices often fold on timescales between 100 ns and 1 μs,^237−240^ and hairpins require milliseconds to fold.^241−243^ Long-range conformational rearrangements in IDPs can occur on timescales between microseconds and milliseconds^88,244^ and possibly even seconds.^189^ Upon liquid–liquid phase separation of IDPs into droplets and condensates *in vitro* and into membraneless compartments in cells, the motions of IDPs are strongly changed. Both translational diffusion of the IDP as well as reorientational dynamics inside the IDP molecule change on a wide range of time and length scales upon phase separation into protein-dense compartments. Because motions of and in molecules play an important role in biochemical reactions, detailed insight into LLPS-induced changes in IDP motions are required.

While insight into the conformational dynamics of IDPs in liquidlike droplets and condensates is still in its infancy, the so far available studies indicate that both local backbone motions and segmental chain motions are decelerated upon LLPS. The LLPS-induced slowing down of conformational dynamics in IDPs on multiple time scales is detected through increased heteronuclear NOE and *R*_2_ values in ^15^N NMR relaxation measurements with residue-specific precision. *R*_2_ spin relaxation rates increased by a factor of ∼3–5 from ∼5 s^–1^ to ∼15–25 s^–1^ upon LLPS at 25–40 °C in different IDPs.^81,83,85^ An exception is the LC domain of hnRNPA2, in which the *R*_2_ rates did not change strongly upon LLPS.^84^ Because of the high temperature at which the later studies were done, the *R*_2_ rates in hnRNPA2 might be dominated by solvent exchange and therefore would not actually probe chain dynamics. These studies, however, lack the analysis of the relaxation rates in terms of time scales and amplitudes of different IDP chain motions, for which more rates are needed. Indeed, LLPS may not only slow down time scales of IDP motions but also restrict the amplitude of the local backbone conformational sampling. Chain entropy reduction inside condensates is indeed predicted by LLPS theory. However, to which proportions the entropies related to longer-range chain motions and to local backbone conformational sampling are affected is not clear.

**Figure 22:**
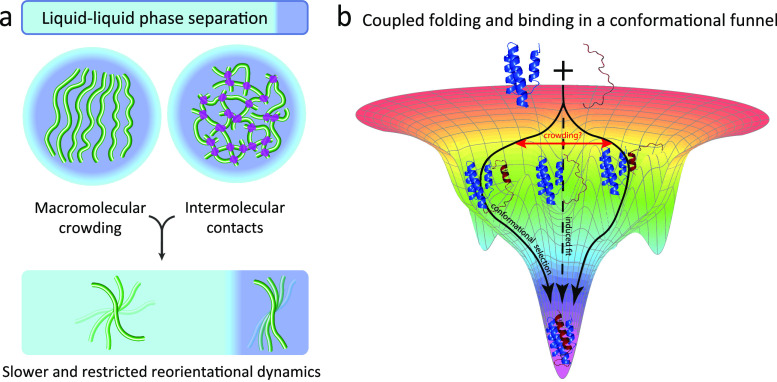
Physico-chemistry of the environment inside membraneless compartments influences IDP dynamics and biochemical reactions with their partners. (a) Molecular crowding and intermolecular interactions that promote liquid–liquid phase separations are expected to slow IDP chain motions in condensates. (b) The free energy landscape of the coupled folding and binding in IDPs may be represented as a conformational funnel.^245^ Different interaction mechanisms, such as induced fit or conformational selection, can be seen as different trajectories along this energy landscape. The reaction can follow multiple trajectories simultaneously, with their relative weights being defined by the shape of the funnel, which may be altered inside membraneless compartments. The presence of macromolecular crowding was, for example, suggested to favor conformational selection-type interactions over induced fit-type interactions.^246,247^ Adapted in part from ref (60) under the terms of the CC-BY 4.0 license.

The exact origin of the slowing and restriction of conformational dynamics inside condensates is not entirely clear. Molecular crowding, the presence of weak nonspecific intermolecular contacts and a resulting increase in the viscosity experienced by IDP molecules will result in slower IDP dynamics inside condensates (Figure 22). This effect might be mimicked by exposing IDPs to high concentrations of crowding agents without inducing a phase separation. Different motions of and in IDP molecules probe the solution viscosity at different length scales and would experience different degrees of slowing in a crowded solution.^168^ Local backbone motions involving individual or a few protein residues are less slowed down upon crowding when compared to chain segmental motions involving multiple residues.^172^ Translational diffusion of IDPs, which happens on the scale of micrometers, is even more slowed down than chain dynamics, for which the relevant length scale is in the nanometer regime.^113^ Importantly, crowding might not have a significant impact on chain conformational sampling, as the amplitudes of motions have been reported to remain almost constant.^113^ However, high protein concentrations inside droplets can cause a transition to the semidilute regime, where protein molecules penetrate each other’s hydrodynamic volumes, which can restrict chain flexibility.

In addition, LLPS is promoted by specific intermolecular contacts (Figure 22). For example, hydrophobic interactions were found to stabilize condensates of domains of elastin-like peptides, yeast polyA-binding protein Pab1,^248^ and FUS LC.^82^ Intermolecular contacts can impact chain dynamics both in terms of timescales and amplitudes of motions. These contacts may be responsible for higher transverse relaxation rates inside Ddx4 condensates compared to a solution of a nonphase separating Ddx4 mutant at a similar concentration^81^ as well as for the increased amplitude of slower motions inside α-synuclein droplets,^59^ both consistent with decreased chain conformational entropy upon phase separation. In addition, as the chain dynamics in IDPs are coupled with the solvent, differences in solvent properties between the dispersed phase and the condensate might play a role, potentially affecting IDP dynamics and their ability to interact with other molecules. A decrease in the solvent ionic strength inside the condensate might, for example, lead to more restricted IDP chain dynamics.^157^

The particular physico-chemistry of the environment inside membraneless compartments influences biochemical reactions that occur inside membraneless compartments. The law of mass action predicts that the reaction rate is proportional to the concentration of the involved molecules, which is significantly increased inside membraneless compartments compared to the outside solution. Accelerated reaction rates were observed, for example, in the carboxylation of ribulose bisphosphate (RuBP) by the Rubisco (ribulose biphosphate carboxylase/oxygenase) enzyme^249−252^ and in the function of the cyclic guanosine monophosphate-adenosine monophosphate (cGA MP) synthase,^253−255^ with mutations impeding LLPS resulting in both reactions being slowed down. The increase in concentration inside membraneless compartments is especially critical in cases where the reaction is rate-limited by the nucleation process and requires a concentration of substrate above a certain threshold, as in case of microtubule assembly from the αβ-tubulin dimers.^256^ Biochemically reconstituted membraneless compartments containing the centrosome proteins SPD-5, PLK1, SPD2, ZYG9, and TPXL1 were found to accelerate microtubule nucleation,^257^ and membraneless compartments containing TPX2 protein were found to accelerate nucleation of new branches of microtubules.^258^ In both cases, this acceleration correlated with the enrichment of condensates in the αβ-tubulin, suggesting that this increase in concentration was the key element in the increase of assembly rates.

Chemical reactions of IDPs that are present in many membraneless compartments are also impacted by the specificities of their environment. The temperature-dependent LLPS of the disordered mitotic spindle protein BuGZ was found to promote microtubule assembly from the spindle matrix and the assembly of both the spindle and the spindle matrix,^259^ potentially involving the nucleation threshold mechanism mentioned above for centrosome proteins and TPX2 protein. Higher concentrations, however, are not the only change inside membraneless compartments compared to the outside solution. Macromolecular crowding inside these compartments may result in excluded volume effects, restricted diffusion, higher effective viscosities for probes of length scales higher than nanometers, and intermolecular interactions all impacting chemical reactions, similar to what is observed and expected in the crowded conditions in living cells.^168^ Zosel et al. probed the thermodynamics and kinetics of binding between the intrinsically disordered activation domain of the steroid receptor coactivator 3 (ACTR) and the molten-globule-like nuclear coactivator binding domain of CBP/p300 (NCBD) in the presence of crowding agents using FRET and fluorescence correlation spectroscopy.^260^ Depletion interactions resulting from an entropic exclusion of the crowding agents in the vicinity of protein molecules were found to stabilize the binding reaction, with the stabilization being more important for larger crowding agents and higher concentrations thereof. The binding reaction rate was experiencing an initial acceleration with increased crowding agent concentrations, which was explained by the effect of the depletion interaction. At higher crowding agent concentrations, the viscosity started to play an important role, slowing down protein diffusion and resulting in binding rate deceleration.

The interaction between folded enzymes and their partners often can be explained using a “lock-and-key” model. Flexibility endows IDPs with a multitude of interaction mechanisms that were described using concepts such as folding-upon-binding, conformational selection, fly casting, and the formation of dynamic complexes.^62−66^ In reality, the interaction mechanisms of IDPs are likely to integrate multiple interaction pathways (as induced fit or conformational selection) at the same time and thus require more complex and broad descriptions, such as the “conformational funneling”.^245^ Changes in the solvent viscosity experienced by the IDP chain inside membraneless compartments, as we discussed in this review, have an impact on the chain dynamics, slowing it down. It was proposed that in crowded environments, conformational selection would be preferred because of the longer contact time between interacting molecules due to slower diffusion.^246^ On the contrary, in the less crowded environment, a faster conformational sampling of interacting molecules could be expected. In enzymes, faster conformation sampling was associated with promoting interactions along the induced fit model.^247^ Local and long-range chain motions are impacted to a different degree due to the length-scale viscosity dependence. Therefore, interaction mechanisms that rely upon, for example, the formation of local secondary structure elements or need long-range chain reconfiguration (such as fly casting motions) are also experiencing a different impact of crowding. Inside membraneless compartments, motions of IDP chains are modulated not only by crowding but also by intermolecular interactions that stabilize LLPS. We therefore suggest that the alteration of dynamic properties of IDPs inside these compartments should lead to the change of relative weights of different interaction pathways between IDPs and their partners, potentially changing the interaction mechanism (Figure 22).

In addition, new mechanisms of interactions involving IDPs and their partners could appear inside LLPS-driven condensates and membraneless compartments. For example, intermolecular interactions that drive LLPS inside condensates result in the formation of system-spanning networks of IDP molecules.^178^ The partner of the IDP in question will therefore interact not with individual IDP molecules but with their networks instead. For example, Nott et al. observed that membraneless organelles formed by the disordered N-terminal domain of Ddx4 can melt double-stranded nucleic acids entering them. This behavior was explained not only by the competition between Ddx4–Ddx4 and Ddx4–DNA/RNA cation−π interactions but in addition by the distortion exerted by the double-stranded nucleic acids on the mesh of interconnected Ddx4 molecules, with the mesh trying to “break” the interfering double-stranded DNA/RNA to regain its nondistorted form.^3^ The dynamics of Ddx4 molecules constituting this “mesh” can, in our opinion, modulate both the thermodynamics and kinetics of the DNA/RNA melting process.

The dynamics of IDPs inside condensates therefore merit detailed studies not only per se but also in the context of understanding biochemical reactions between IDPs and their partners under these conditions. Multifield NMR spin relaxation measurements analyzed in terms of characteristic times and amplitudes of individual motional modes can provide a framework for understanding the impact of LLPS and biomolecular condensation on IDP dynamics on the picosecond-to-nanosecond time scale. These methods have already proven very powerful in the characterization of the monomeric dispersed phase of IDPs.^69,131,135,172^ They provide quantitative and residue-specific information about the chain flexibility, the presence of nascent secondary structure elements, as well as the formation of hydrophobic clusters in IDPs, all important for understanding the process of phase separation and of the interactions in which IDPs are involved inside condensates.

Additionally, the crowding effect of LLPS might be modeled through the study of dynamic properties of IDPs in highly viscous, nonphase separated states.^172^ The characteristic times and amplitudes of motions that are derived from the crowded, nonphase separated state can then be compared with values obtained in the actual IDP condensates. With NOE and PRE^44,81^ measurements performed to identify intermolecular contacts inside condensates, the comparison of amplitudes and timescales could then be used to quantify the impact of these contacts on chain conformational sampling and timescales of dynamics. In particular, changes in motional amplitudes, not observed in IDP crowding experiments, in IDP regions where intermolecular contacts were identified, could be pointing at a reduction of chain conformational entropy due to the formation of these contacts. Combined with relaxation dispersion NMR measurements to probe exchanging nonvisible sparsely populated conformations potentially more involved in intermolecular contracts, multifield NMR spin relaxation measurements, NOE and PRE measurements promise to delineate the impact of intermolecular interactions on IDP dynamics in condensates at single residue resolution.

Information about IDP dynamics on longer time scales, between nanoseconds and milliseconds, can be provided by fluorescence correlation spectroscopy. Unlike NMR methods, which provide ensemble-averaged dynamics parameters of IDPs, fluorescence correlation spectroscopy^161^ and fluorescence anisotropy^189^ can be performed in a single-molecule setup, potentially providing information on different conformational populations of IDPs and their role in the formation of intermolecular contacts inside condensates. Given the broad dynamics timescale range and conformational landscape of IDPs, it will be important to combine NMR studies with nanosecond fluorescence correlation spectroscopy, EPR spectroscopy, and fluorescence anisotropy experiments to fully understand the impact of phase separation on the conformational dynamics in IDPs.

Understanding the dynamics of transient and sparsely populated conformational states and their involvement in intermolecular interactions is of key importance to understand the mechanisms of interactions between IDPs and their partners inside condensates, as they can involve the funneling of multiple initial IDP conformations and the formation of encounter complexes. Population-specific information about structural and dynamic changes inside condensates can furthermore be gained through molecular dynamics simulations.^110,198,219,221,261,262^ All-atom MD simulations of IDPs inside condensates are still limited by the huge computational cost of modeling high numbers of densely packed molecules. In addition, even in MD simulations of single IDP molecules, the reproduction of residual secondary structure elements has not been perfect, reducing their effectiveness at describing IDP dynamics, where secondary structure elements are often crucial. Coarse-grained simulations of IDPs inside condensates are less computationally demanding but are also not perfect in reproducing the secondary structure elements in IDPs. However, with the current pace of hardware development, there is a hope that in the near future all-atom simulations will be more and more feasible and their number will be increasing. Better knowledge of the coupling between protein and water dynamics in condensates would help us to improve water models and force fields and would likely improve the prediction of time scales of IDP chain motions. Coarse-grained simulations, on the other hand, could benefit from increasing experimental evidence about IDP behavior in condensates. Additionally, improvements aimed at reproducing secondary structure in all-atom simulations could be translated to coarse-grained models. Combined with NMR, EPR, and fluorescence spectroscopy methods, MD simulations will be able to provide us a detailed, and consistent with experimental data, picture of molecular interaction mechanisms of IDPs related to LLPS and of IDP-associated chemical reactions in biomolecular condensates.
